# Early fate diversification of radial glial progenitors during corticogenesis

**DOI:** 10.1126/sciadv.adw5487

**Published:** 2026-08-05

**Authors:** Irene Varela-Martínez, Ana Villalba, Jorge García-Marqués, Alfonso Aguilera, Diogo S. Castro, Simon Hippenmeyer, Marta Nieto

**Affiliations:** ^1^ Department of Molecular and Cellular Biology, Centro Nacional de Biotecnología, Consejo Superior de Investigaciones Científicas (CNB-CSIC), Madrid 28049, Spain.; ^2^ Institute of Science and Technology Austria (ISTA), Am Campus 1, 3400 Klosterneuburg, Austria.; ^3^ i3S Instituto de Investigação e Inovação em Saúde da Universidade do Porto, Rua Alfredo Allen, 208, 4200-135 Porto, Portugal.

## Abstract

Radial glial progenitors (RGPs) generate all projection neurons (PNs) in the cerebral cortex through incompletely understood processes. We combined Mosaic Analysis with Double Markers at embryonic stages (E)12.5 and E13.5 with early postnatal callosal tracing to dissect RGP lineage progression. We find that multipotent RGPs generate all extra-telencephalic (ET) and intra-telencephalic (IT) PNs via parallel sublineages that emerge simultaneously at neurogenesis onset. ET-PN production progresses exclusively via small, self-consuming lineages; IT-PN lineages feature RGPs generating large translaminar outputs. The early emergence of IT-PN–fated RGPs, coinciding with a switch to direct neurogenesis, contributes to the stereotyped population-level progression of the multipotent lineage. We also identify POU3F transcription factors as candidate regulators of IT-PN fate via noncanonical mitotic chromatin binding. The results support a model whereby IT- and ET-PNs arise from an early bifurcation and parallel specification within the multipotent RGP lineage.

## Teaser

Multipotent RGPs generate cortical neurons via parallel small ET-PN lineages and large translaminar IT-PN–fated RGP lineages.

## INTRODUCTION

The neocortex, a hallmark of mammalian evolution, underlies higher-order cognitive functions. These functions rely on complex cortical networks of excitatory projection neurons (PNs) generated during embryonic development. The production of PNs in the developing cerebral cortex follows an overall inside-out sequence of neurogenesis: Early-born neurons settle into deep cortical layers (DLs), whereas late-born neurons migrate past them into progressively more superficial positions, forming the upper layers (ULs). Radial glial progenitors (RGPs) generate all cortical PNs (*1*, *2*) and serve as primary targets of key mechanisms driving the expansion and increased complexity of the human cerebral cortex (*3*–*5*).

Mosaic Analysis with Double Markers (MADM)–based clonal analysis in mice has provided an inaugural quantitative framework of cortical neurogenesis (*6*–*11*). These studies show that once RGPs enter the neurogenic phase around embryonic day (E) 12.5, a single RGP generates approximately eight to nine PNs distributed across the cortical layers (*8*–*10*), through a combination of both direct and indirect neurogenesis (*12*, *13*). Yet, the precise lineage logic by which RGPs produce the correct numbers and subtypes of cortical PNs remains elusive (*10*). Equally unresolved is the degree of heterogeneity that exists within RGP populations themselves (*8*, *10*, *14*–*19*).

Laminar location has long served as a proxy for neuronal identity. However, neuronal identity is intrinsically complex and multifactorial, requiring the integration of multiple molecular, morphological, and functional features. On the basis of axonal projection patterns, PNs can be broadly classified into two main categories: (i) intra-telencephalic (IT) PNs, present across all layers, which interconnect cortical regions and/or the basal telencephalon (*20*, *21*), and (ii) extra-telencephalic (ET) PNs, which target subcortical areas outside the telencephalon—such as the thalamus, pons, and spinal cord—and are confined to the DLs (*22*–*25*). Notably, ET-PNs and IT-PNs subtypes sometimes coexist within the same cortical layers, raising the question of how their generation is developmentally coordinated.

This conceptual complexity was already foreshadowed by early birth-dating studies, which detected the concurrent generation of some DL-PNs with UL-PNs (*26*). Recent work has shown that synchronically labeled progenitors can yield heterogeneous laminar outputs (*27*–*29*). In particular, using FlashTag labeling and single-cell analyses, Magrinelli *et al.* demonstrated that early apical progenitors (APs) produce progeny that diversify into multiple laminar, molecular, and connectivity-defined types, whereas later-born cohorts destined for the ULs exhibit markedly more homogeneous fingerprints. The authors proposed that, as corticogenesis proceeds, APs are governed by increasingly robust mechanisms that ensure predictable laminar identities (*28*). In parallel, genetic lineage-tracing studies have shown that laminar identity can also emerge via intermediate progenitors (IPs) that generate multiple PN types, even when specified isochronically (*27*, *29*). Collectively, these findings demonstrate heterogeneity in the laminar fates produced during the same temporal windows, adding nuance to the relationship between laminar position and PN subtype.

Projection-based classifications represent a technical challenge as no straightforward tracing method can comprehensively capture the full axonal diversity of adult IT-PNs and ET-PNs. In contrast, developing PNs share exuberant axonal projections that can be leveraged to identify projection subtypes (*30*–*33*). Studies from our laboratory have shown that early in development, and by default, all differentiating layer 2/3 and layer 4 PNs (UL-PNs) initially extend exuberant axons through the corpus callosum (CC) (*33*). Such display of interhemispheric projections coincides with the transcriptional expression of an immature callosal projection neuron (CPN) identity (*34*), both reflecting a transitional stage along the dynamic differentiation program of L2/3 and L4 PNs. With maturation, developmental callosal projections undergo major activity-dependent refinement. Accordingly, these projections are successfully stabilized or eliminated during the first three postnatal weeks, correlating with the acquisition of more definitive PN identities (*33*, *35*). Because UL-PNs purely comprise IT-PN populations, these data together suggest that developmental callosal axons are a common signature of immature IT-PN identity. Notably, a transient CPN molecular profile is also observed in a cluster of DL-PNs (*34*), and numerous PNs in the DLs display developmental callosal axons (*33*). Whether these projections from the DL-PNs identify IT-PN identities has not been examined.

Here, we examined the early axonal projections of differentiating cortical PNs and performed clonal lineage analysis that leverages developmental callosal projections to classify RGP outputs. First, we demonstrated that injections of fluorescent axonal tracers in the CC performed at early postnatal stages distinguish ET- from IT-PNs. Next, by combining early callosal tracing with classical birth-dating strategies, we found that ET-PN and IT-PN populations in the DLs were generated over sequential, only partially overlapping neurogenic periods. In other words, many ET-PNs and IT-PNs are born asynchronously, yet settle within the same layer. These results, in line with previous observations (*27*–*29*), confirm and provide new evidence for heterogeneity in the developmental birthdates of DL-PNs. We identified one of the “sources” of this disparity, suggesting that PN subtypes diversify semi-independently and in parallel with the canonical laminar-temporal patterning.

To investigate the lineage architecture underlying the orderly PN projection–type production, we combined early callosal projection tracing with MADM-based clonal analysis of RGPs. Using this approach, we retrospectively classified *Emx1*-derived MADM clones, labeled at E12.5 or E13.5, based on their early axonal patterns. The analysis revealed that neurogenesis progresses through pure ET-PN and IT-PN sublineages, which emerge directly from the multipotent RGP lineage at the onset of the neurogenic phase, by E12.5. Accordingly, single PN outputs were very infrequent in neurogenic E12.5 RGP clones; instead, we observed outputs consisting of two to three PNs. ET-PN lineages were invariably small (two to three PNs of the same DLs), while IT-PN lineages included both small self-consuming lineages and large translaminar (DL+ UL) subclones that progressed through IT-PN–fated RGPs. Notably, comparing the number and laminar position of the neuronal outputs emerging from the E12.5 MADM population indicated a smaller and slower production of IT-PNs compared to ET-PNs. In this fashion, the simultaneous specification and parallel progression of the sublineages results in asynchrony in birth times. At the same time, the mode of progression of the IT-PN–fated RGPs mediate a final higher number of IT-PNs relative to the number of ET-PNs.

Last, although population-level analyses revealed a stereotyped pattern of lineage progression—most notably the robust conservation of the IT-PN/ET-PN production ratio—the analysis of initial neurogenic divisions could not exclude stochastic contributions to the earliest fate decisions, consistent with previous studies (*15*, *28*). Not every E12.5 neurogenic division produced an ET-PN sublineage from one mitotic cell and an IT-PN sublineage from the other. Instead, in the small outputs, we observed both pure ET-PN and IT-PN sublineages. In the Major subclones, we found an equal frequency of the daughter RGP either acquiring an IT-PN fate or maintaining the multipotent state of the parental E12.5 RGP. This result would agree with variable trajectories for the lineage progression of a multipotent RGP. However, despite the lack of stereotyped divisions in individual clones, distribution analysis showed that population-wise, the early specification of IT-PN–fated RGPs organizes cortical neurogenesis and generates a quantitatively and temporally stereotyped process. Besides, our data suggest that POU3F proteins are involved in the specification of RGPs to IT-PN fate through sequence-independent, noncanonical bookmarking of mitotic chromatin, offering a mechanism for stabilizing fate bias across divisions.

Our work provides direct clonal evidence that an early projection-identity decision manifests in distinct cortical lineage behaviors. Rather than viewing ET and IT diversity solely as an emergent consequence of laminar progression, our data support a process in which the specification of a nascent broad projection-type identity occurs early in the lineage and runs in parallel to the orderly generation of laminar fates. Our model’s main conceptual advance is identifying projection-type specification, coupled with the emergence of parallel sublineages, as an additional organizational axis in the neurogenic progression of multipotent RGPs. In this refined framework, cortical lineage diversity reflects not only differences in laminar sequence, but also divergence into projection-subtype–fated branches within the multipotent progenitor pool.

## RESULTS

### Developmental callosal projections as a specific marker for IT-PNs

**Fig. 1.**
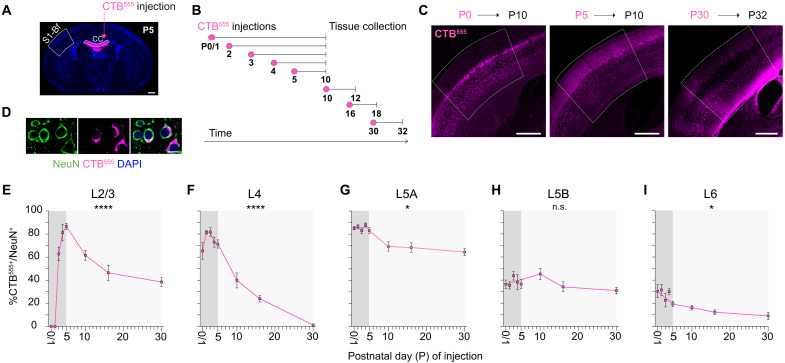
IT-PNs across all cortical layers extend developmental callosal axons. (**A**) CTB-555 signal confined within the CC 2 hours postinjection. (**B**) Experimental paradigm for CPN analysis: Saturating CTB injections into the CC were performed (i) at P0/1, P2, P3, P4, or P5 and brains were analyzed at P10, once neuronal migration is completed, or (ii) at P10, P16, or P30, and brains were analyzed 48 hours after the injection. (**C**) CTB-555 signal in the S1-Bf cortex contralateral to CC injection sites at P0 (left), P5 (middle), and P30 (right). The stage shown in magenta indicates the time of injection, and the stage shown in black indicates the time of analysis. (**D**) High-magnification image of L6 PNs labeled for NeuN (green), CTB-555 (magenta), and DAPI (blue) from P5-injected brain analyzed at P10. (**E** to **I**) Layer-specific dynamics of callosal axonal elimination across developmental stages. Animals were injected and analyzed as in (B). The graph represents the percentages of CPNs (CTB^+^NeuN^+^) per cortical layer (*y* axis) and time of injection (*x* axis). Mean ± SEM (*n* = 500 cells, *n* = 2 sections per animal; *n* ≥ 4 animals per stage). One-way ANOVA (CTB^+^NeuN^+^ versus Time_P5-P30_): (E) ****P < 0.0001, (F) ****P < 0.0001, (G) *P = 0.0223, (H) *P* = 0.1399 (n.s.), and (I) *P = 0.038. In (H) and (I), the curve was fitted between P0/1 and P5 to illustrate the temporal dynamics more effectively. Scale bars, 500 μm [(A) and (C)] and 10 μm (D).

To determine whether early callosal projections specifically characterize IT-PNs in the ULs and DLs, we investigated their interhemispheric projections using injections of fluorescently labeled retrograde tracer molecules [cholera toxin subunit B (CTB) fluorescent conjugates] into the CC (Fig. 1A). Unlike cortical plate injections, this method saturates the callosal tract and enables quantification of the absolute number of PNs with developmental or mature callosal axons. To examine axonal refinement across differentiation, we injected the animals at multiple time points ranging from postnatal day (P) 0 to P30 (Fig. 1, B and C). A detailed description of the methodology, including validation of specificity, saturation, and efficacy, has been previously reported (*33*). For the analysis, we focused on the primary somatosensory cortex (S1). For quantification, to avoid the diluting effects of nonneuronal cells such as glia, we analyzed CTB cells labeled for the neuronal marker NeuN (CTB^+^NeuN^+^ cells) (Fig. 1D and fig. S1A). In the ULs, the high proportion of CTB labeling between P0 and P5 (Fig. 1, E and F, and fig. S1, A and B) was consistent with the previous demonstration of the common early CPN identity of L2/3 and L4 (*33*–*36*).

In the DLs, the experiment provided unprecedented precision in the definition of the proportion of PNs with developmental and mature CPN identity (Fig. 1, G to I, and fig. S1, A to C). Quantifications showed that L5A is principally composed of CPNs, which, unlike UL-PNs, undergo minimal pruning from birth to adulthood (Fig. 1G). Besides, approximately 30% of the PNs in L6 and L5B exhibited early postnatal callosal projections (Fig. 1, H and I). In L5B, CPN numbers remained stable from P0 to P30 (Fig. 1H), while in L6, postnatal pruning diminished the proportion of adult CPNs to about 10% (Fig. 1I).

We additionally investigated the connectivity with the dorsal striatum—a telencephalic structure that, alongside the cortex, receives inputs from PN subpopulations, both developmentally and in the adult (*37*, *38*). To this end, we performed dual injections into the CC and the opposite dorsal striatum. This approach examines PNs projecting to the contralateral striatum via the CC, as well as those with a dual projection pattern targeting the ipsilateral striatum and the contralateral hemisphere through the CC (fig. S1, D to J). Significant fractions of L5A and L5B PNs showing developmental callosal axons were labeled from the striatum. This pattern was also observed in the other layers in more minor subsets of PNs (fig. S1J). Thus, developmental CPN identity includes a subset of IT-PNs with projections to the striatum.

**Fig. 2.**
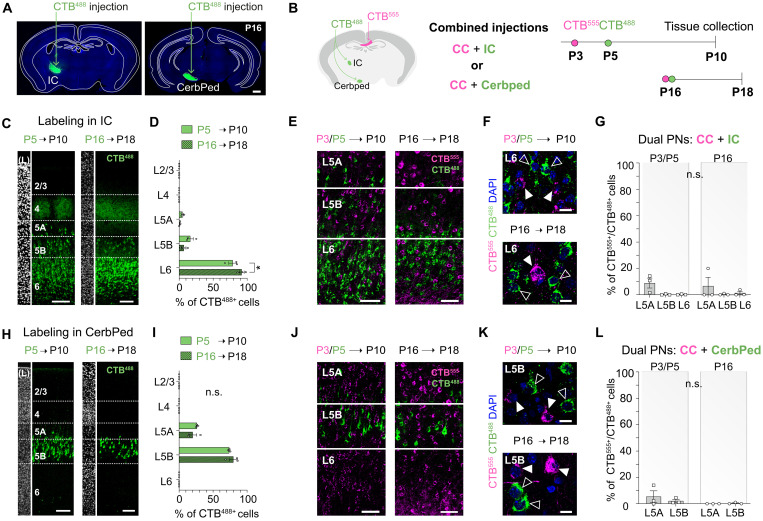
Early postnatal axonal tracing from the CC differentiates IT-PNs from ET-PNs. (**A**) CTB-488 signal confined within the IC (left) or the CerbPed (right) 2 hours postinjection. (**B**) Injection scheme: sequential (P3 and P5) or simultaneous (P16) injections into the CC (magenta) and the IC or the CerbPed (green) to analyze dual-projection neurons. (**C** and **H**) CTB-labeled S1-Bf PNs projecting through the IC (C) or the CerbPed (H) following injections as in (A). (**D** and **I**) Percentage of CTB-488^+^ cells (ET injection) per cortical layer after injections as in (A). Layer distributions are calculated from the total number of CTB-488^+^ cells in S1-Bf area sections. Mean ± SEM (*n* = 2 sections per animal; *n* = 3 animals per stage and type of ET injection). Two-way ANOVA with Šidak’s post hoc test (CTB^+^ Time × Layer): *P value_L6 P5 versus L6 P16_ = 0.0147 in (D). (**E** and **J**) CTB-488 (green, ET-PNs) and CTB-555 (magenta, IT-PNs) signals segregated into different PNs after injections as in (B). (**F** and **K**) High-magnification images from [(E) and (J)] CTB-488^+^ (green, empty arrowheads) and CTB-555^+^ cells (magenta, white arrowheads). (**G** and **L**) Percentage ratio of CTB-555^+^ PNs over CTB-488^+^ PNs after IC (G) or CerbPed (L) injections as in (B). Percentages are calculated from the total number of CTB-488^+^ cells quantified in the area occupied by L5A, L5B, or L6 across S1Bf. Mean ± SEM (*n* = 2 sections per animal; *n* = 3 animals per stage). The two-way ANOVA analysis for comparison with a theoretical zero showed nonsignificant main effects or interactions. Scale bars, 500 μm (A), 100 μm [(C) and (H)], 50 μm [(E) and (J)], and 10 μm [(F) and (K)].

We next investigated whether DL-PNs with developmental callosal axons might express a hybrid identity and also extend projections to ET territories. To address this, we conducted a series of experiments combining injections into the CC with subsequent injections into either the internal capsule (IC) or the cerebral peduncle (CerbPed). We chose these two anatomical sites as they harbor axons of the major ET projection routes, namely, the corticothalamic, corticopontine, and corticospinal fibers (*39*). One group of animals received CC injections at P3 followed by IC or CerbPed injections at P5, while another group received both injections at P16 to assess a more advanced stage of axonal refinement (Fig. 2, A and B).

Both types of ET injections demonstrated specificity, labeling cells exclusively in the DLs. Across postnatal development, the layer distributions of cells labeled from ET territories remained largely consistent between P5 and P16 injections, indicating limited pruning during this interval (Fig. 2, C, D, H and I). Notably, the vast majority of labeled cells were single-positive, i.e., labeled exclusively by either from the CC or ET injection (Fig. 2, E to G and J to L, and fig. S1, K and L). The results indicate that ET-PNs do not project inter-hemispherically, neither during development nor in adulthood. Moreover, we confirmed that expression of CTIP2, a marker of ET-PN identity, correlates with the absence of developmental callosal projections (fig. S1, M and N). Altogether, the experiments demonstrate that early callosal projections are a reliable indicator of early immature IT-PN identities, not only for UL-PNs but also for DL-PNs. This distinction provides a valuable criterion for the prospective identification of the major PN subtypes in the neocortex.

### Partially overlapping sequential production of ET and IT PNs

**Fig. 3.**
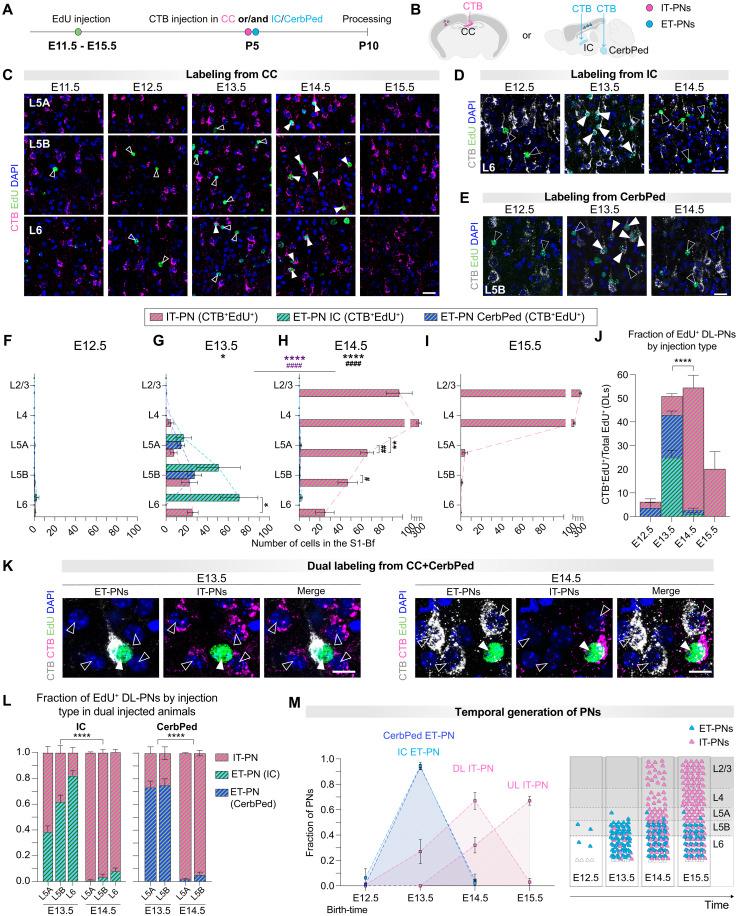
Temporal offset in the generation of IT-PNs and ET-PNs of the DLs. (**A**) Experimental design. (**B**) CTB injection sites. (**C** to **E**) Representative images of DL-PNs traced from the CC (C), IC (D), or CerbPed (E). Empty arrowheads: EdU^+^, white-filled arrowheads: CTB^+^ EdU^+^. (**F** to **I**) Birth-dating analysis of IT-PNs and ET-PNs (IC and CerbPed). Graphs show number of CTB^+^EdU^+^ cells per layer. Three-way ANOVA (purple) _E13.5 versus E14.5 × IT-PN versus ET-PN_: ****P_IT-PN versus ET-PN IC_ < 0.0001 and ^####^*P*_IT-PN versus ET-PN_-_CerbPed_ < 0.0001. Two-way ANOVA (CTB^+^EdU^+^ across PN subtypes): *P_E13.5 IT-PN versus ET-PN-IC_ = 0.0363, ****P_E14.5 IT-PN versus ET-PN-IC_ < 0.0001, and ^####^*P*_E14.5 IT-PN versus ET-PN-CerbPed_ < 0.0001. Šidak’s post hoc test: (E13.5) *P_L6 IT-PN versus L6 ET-PN IC_ = 0.0396; (E14.5) **P_L5A IT-PN versus L5A ET-PN-IC_ = 0.0065, ^#^*P*_L5B IT-PN versus L5B ET-PN-CerbPed_ = 0.00331, and ^##^*P*_L5A IT-PN versus L5A ET-PN-CerbPed_ = 0.0016. (**J**) Stacked fractions of DL-IT-PNs and ET-PNs from successive neurogenic waves. One-way ANOVA with Tukey’s post hoc test (DL-IT-PNs-CTB^+^EdU^+^ across consecutive stages): ****P_E13.5 versus E14.5_ < 0.0001. (**K**) Images of L5B PNs traced from the CC and CerbPed with EdU at E13.5 (left) and E14.5 (right). Empty arrowheads: CTB^+^EdU^−^, white-filled arrowheads: CTB^+^ EdU^+^ cells. (**L**) Fraction of EdU^+^ DL-PNs by injection type in dually injected animals. Two-way ANOVA with Šidak’s post hoc test (CTB^+^EdU^+^ versus Time, per PN-subtype and layer): ****P_E13.5 versus E14.5_ < 0.0001 in all layers and PN-subtypes. (**M**) Estimated fraction of PN subtypes born at successive embryonic stages (left) and schematic of sequential ET-PN/IT-PN neurogenesis (right). [(C) to (E) and (K)] CTB-magenta (IT-PNs), CTB-gray (ET-PNs), EdU-green, and DAPI-blue. [(F) to (J) and (L)] Mean ± SEM (*n* = 2 sections per animal; *n* ≥ 3 animals per stage/type of CTB injection or per stage × group). Scale bars, 20 mm [(C) to (E)] and 10 mm (K).

The experiments showed that retrograde tracing from the CC at early postnatal stages specifically distinguishes IT-PNs from ET-PNs and serves to discriminate the two types in the DLs. To examine the birth times of IT-PN and ET-PN subtypes, we administered single doses of 5-ethynyl-2′-deoxyuridine (EdU) to pregnant dams at embryonic day (E) 11.5, 12.5, 13.5, 14.5, or 15.5. In the first set of experiments, one group of EdU-administered animals was injected with CTB into the CC to elucidate the birthdates of IT-PNs; two other groups of pups were injected into either the IC or the CerbPed to examine the birthdates of ET-PNs. All types of injections were performed at P5, a stage preceding major callosal refinement (Fig. 3, A and B).

We then quantified the number of CTB^+^EdU^+^ double-labeled cells (newborn IT-PNs or ET-PNs) across all layers of the S1-barrel field (Bf) cortex at P10, when both migration and developmental cell death are finalized (*33*, *40*) (Fig. 3, C to E). For the analysis, to compare the layer position and the magnitude of the ET-PN and IT-PN outputs across embryonic stages, we plotted the number of CTB^+^EdU^+^ cells per S1-Bf area section (Fig. 3, F to J, and fig. S2, A to E).

EdU birth-dating aligned with classical studies, showing that neuronal output was lower at E11.5 and E12.5 compared to later stages, and confirming the overall inside-out organization across neurogenic waves (fig. S2, A and B). However, when analyzed by projection type, the birth of IT-PNs in the DLs exhibited a clear delay relative to the generation of ET-PNs, which were labeled from the IC or the CerbPed and showed a sharp neurogenic wave at E13.5 (Fig. 3, F to I).

A significant number of DL-IT-PNs were labeled with EdU injections at E14.5, which also labeled L4 PNs (Fig. 3H). In absolute numbers, E14.5 DL-IT-PNs represented a fraction almost equal to E13.5 ET-PNs (Fig. 3, G and H). With the combined data of all the injected animals, we obtained an estimate of the projection types that comprise the sequential neurogenic waves populating the DLs. This reflected a clear segregation of ET-PNs at E13 and of IT-PNs at E14.5. Only a small fraction of the E13.5 output were IT-PNs (Fig. 3J). As a control, we confirmed that the E14.5 EdU^+^ cells that we labeled from the CC in the DLs appropriately express NeuN (CTB^+^EdU^+^NeuN^+^), as expected, and thus that the results were not artifactual due to the labeling of nonneuronal populations (fig. S2, F to H).

Notably, E12.5 outputs were not labeled from the injections in the CC or from ET (IC or CerPed) injections (Fig. 3F), indicating that they represented a DL subpopulation that do not extend callosal projections or long subcortical projections, such as the TLE4^+^ L6 PN subpopulation (*41*). Although our injections do not account for this population of E12.5 ET-PNs, their lack of callosal projections identifies them as non–IT-PNs, thereby supporting the conclusion that CC injections selectively label IT-PNs. We analyzed the E13.5 and E14.5 cohorts with injections for ET-PNs and IT-PNs in the same animal using two different fluorescent conjugates of CTB (Fig. 3K). ET-PNs were only found in E13.5 cohorts, and reciprocally, E14.5 EdU^+^ cohorts marked the birth of IT-PNs and not ET-PNs in the same layer. As in single CTB injections, the major fractions of ET-PNs were found in L6 and L5B, while the outputs that migrate to L5A comprised predominantly IT-PNs (Fig. 3L). These experiments discard results due to developmental variability among the pups and confirmed that a neurogenic burst of ET-PNs at E13.5 precedes the birth of most IT-PNs, including those that will populate the DLs. Subsequently, with the results of the single injections combined, we estimated the timeline of ET-PN and IT-PN birth across development (Fig. 3M and fig. S3). For individual layers, the analysis indicated that half of L6 IT-PNs are born at E13.5 and half at E14.5, most layer L5B IT-PNs are born at E14.5, and the birth of layer L5A IT-PNs is tightly confined to E14.5 (fig. S3).

Next, to further assess a sequential birth of ET-PNs and IT-PNs using a complementary experimental approach, we performed in utero electroporation (IUE) experiments targeting E13.5, E14.5, and E15.5 ventricular progenitors (fig. S4, A to D). The analysis of the patterns of the axonal projections of their PN outputs confirmed the narrow window for ET-PN production. We observed green fluorescent protein (GFP)^+^ axonal projections in the IC and thalamus exclusively in E13.5 outputs (fig. S4E and S4F), whereas in animals electroporated at E14.5 and E15.5, we found GFP^+^ axons in the CC and the striatum (fig. S4, G to J).

Together, these results demonstrate that only a subset of DL-IT-PNs is generated synchronously with ET-PNs. Notably, in both ET- and IT-PN lineages, we observe a clear inside-out temporal sequence of layer generation, indicating a superimposition of projection and laminar identities. These findings suggest that neurogenesis proceeds hierarchically, with the specification of projection subtypes occurring in parallel with the sequential acquisition of layer identities.

### IT-PN–fated RGPs emerge from E12.5 multipotent RGPs

**Fig. 4.**
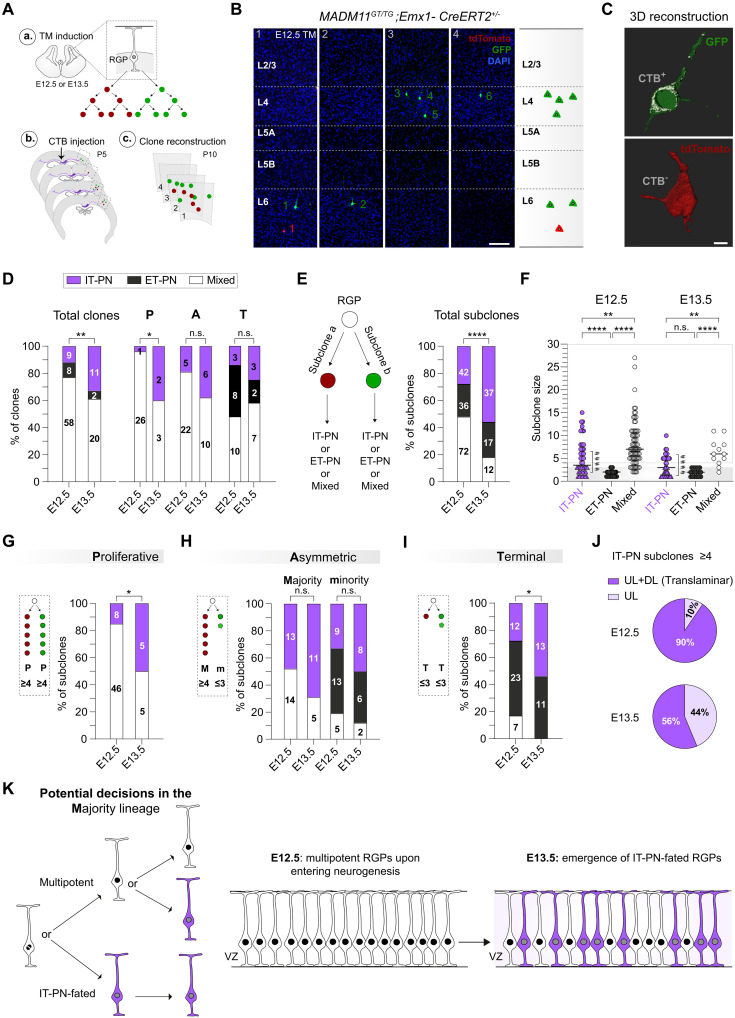
IT-PN–fated RGPs emerge from E12.5 MADM-labeled divisions. (**A**) Experimental design. (**B**) Illustrative images of an asymmetric clone (panels 1 to 4) composed of a red (tdTomato) and a green (GFP) subclone. Clone reconstruction (rightmost panel). (**C**) 3D Imaris reconstruction of a CTB^+^ IT-PN (GFP in green, CTB in white) and a CTB^−^ ET-PN (tdTomato in red). (**D**) Proportion of IT-PN, ET-PN, and Mixed clones at E12.5 and E13.5. Chi-square test_E12.5 versus E13.5_: (Total clones) **P = 0.0086, (**P**roliferative) *P = 0.0105, (**A**symmetric) *P* = 0.1679, and (**T**erminal) *P* = 0.4427. (**E**) (Left) Subclone definition and classification. (Right) Classification of E12.5 and E13.5 subclones. Bold numbers represent the number of subclones. Chi-square test_E12.5 versus E13.5_: ****P < 0.0001. (**F**) Size of MADM subclones. E12.5, *n* = 150; E13.5, *n* = 66. Bar represents the median. Kruskal-Wallis test with Dunn’s post hoc test: (E12.5) ****P_IT versus ET_ < 0.0001, **P_IT versus Mixed_ = 0.0022, and ****P_ET versus Mixed_ < 0.0001; (E13.5) (n.s.) *P*_IT versus ET_ = 0.2066, **P_IT versus Mixed_ = 0.0049, and ****P_ET versus Mixed_ < 0.0001. Kolmogorov-Smirnov test comparing IT-PN sizes [small (≤3) versus large (≥4)]: ^####^*P* < 0.0001. (**G** to **I**) Subclones by size and PN types. Chi-square test: (G) *P= 0.0111, (H) (Majority) (n.s.) *P* = 0.1885, (minority) (n.s.) *P* = 0.5523, and (I) *P= 0.0392. (**J**) Fraction of translaminar versus pure-UL in the IT-PN sublineages of size ≥4. (**K**) Schematic of key observations: Neurogenic divisions of E12.5 multipotent RGPs produce either IT-PN-RGPs or multipotent RGP sublineages in similar frequencies. As a consequence, the proportion of IT-PN-RGPs increases between E12.5 and E13.5. Color code [(D) to (I)] Clone/subclone types: IT-PN (purple), ET-PN (black), and Mixed (white). In bold inside the bars, total number of subclones or clones of that category. Scale bars, 100 μm (B) and 5 μm (C).

Our analysis indicated that projection-subtype specification is a primary neurogenic decision and suggested a sequential origin of ET-PNs and IT-PNs within progenitors’ lineage progression. The result also showed that, for DL-PNs, layer position informs poorly of their birthdates and position within the neurogenic lineage. To investigate how individual RGPs specify and produce IT-PNs and ET-PNs, we combined our CTB injection protocol with MADM clonal analysis (*6*, *42*). We used the *MADM-11*^GT/TG^; *Emx1-CreERT^2^* line to induce sparse labeling of dividing VZ progenitors following TM administration at E12.5 or E13.5 (Fig. 4A and fig. S5A). EdU birth-dating experiments confirmed that neurogenesis of IT-PNs follows the same temporality and is indistinguishable in the MADM (*11*^GT/TG^; *Emx1*) and WT (BL6C57) mice (fig. S5B).

For IT-PN identification, we performed saturating injections of CTB into the CC at P5. We then individually assessed CTB labeling in each MADM-labeled cell at P10 (Fig. 4, A to C). We analyzed 75 clones labeled by TM inductions at E12.5 and 33 clones at E13.5, all in the S1-Bf cortex. On the basis of subclone sizes, we inferred the division pattern of individual progenitors classifying MADM clones as proliferative (two subclones ≥4 PNs), asymmetric (majority subclone ≥4 PNs and minority subclone ≤3 PNs), and terminal (two subclones of ≤3 PNs) (fig. S6, A to F). Proliferative and asymmetric divisions identify RGP divisions, while terminal divisions may not. The raw data of the MADM analysis are provided in the accompanying supplementary dataset and annotated atlas (see the Supplementary Materials). MADM^+^ cells and MADM^+^CTB^+^ cells showed the expected cell death (fig. S6G), layer distributions (fig. S6H), and DL/UL ratios (fig. S6I) in accordance with previous studies (*8*). Quantifications confirmed that the CTB labeling efficiency of MADM outputs was comparable to that of WT animals (fig. S6J). All UL-PNs were classified as IT-PNs, while DL-PNs were distinguished as IT-PNs (CTB^+^) or ET-PNs (CTB^−^).

Seventy-seven percent (58 out of 75 clones; i.e., 58/75) of the clones produced from E12.5 MADM divisions include both IT-PNs and ET-PNs (Mixed clones). Specifically, 96% (26/27) of the proliferative clones and 81% (22/27) of the asymmetric clones were Mixed clones (Fig. 4D). Hence, in overall agreement with previous studies, we confirmed that the E12.5 RGP population is multipotent (*8*, *10*). From here on, we refer to multipotent RGPs as those RGPs producing both ET-PNs and IT-PNs. Besides Mixed clones, 23% (17/75) of E12.5 clones were pure ET-PN (11%, 8/75) or IT-PN (12%, 9/75) lineages. Notably, pure ET-PN clones were exclusively produced from terminal divisions (Fig. 4D). For these clones, we cannot discard the fact that they represent other types of *Emx1*-*Cre* labeled progenitors and not RGPs, such as *Emx*1^+^ IPs (*11*). Furthermore, from E12.5 to E13.5, the proportion of ET-PN clones remained low, also indicating the lack of RGP-mediated expansion. Thus, our data do not support the existence of ET-PN–fated RGPs, except possibly in the case of a low proliferative subtype.

The 12% of E12.5 pure IT-PN clones were found across all types of divisions—proliferative, asymmetric, and terminal divisions. Their frequency was very low (≈4%, 1/27) in proliferative clones but constituted around 23% (5/22) of the asymmetric clones. At E13.5, their frequency increased significantly in all categories (Fig. 4D). These results indicated a lineage progression through the early specification of RGPs to IT-PN fates. Statistical analysis confirmed that such prevalence of IT-PN pure clones could not be explained by the death of ET-PNs in Mixed clones (see Materials and Methods). Hence, MADM demonstrates the early emergence of IT-PN–fated RGPs.

Next, we investigated the neurogenic decisions underlying the progression of E12.5 multipotent RGPs. To this end, we analyzed potential decisions in the daughter cells and in the separate subclones (hereafter referred to as sublineages) (Fig. 4, E to I). Approximately half of the sublineages emerging from the E12.5 RGP population were Mixed sublineages (72/150). Among the other half, there were almost as many ET-PN sublineages (36/150) as IT-PN (42/150) sublineages. From E12.5 to E13.5, the proportion of IT-PN sublineages increased at the expense of decreases in multipotent sublineages with no changes in ET-PN sublineages (Fig. 4E). Hence, ET-PN and IT-PN sublineages are specified early (around E12.5), simultaneously and in similar frequencies, but only IT-PN sublineages progress through RGPs. In agreement, as with the clones, ET-PN sublineages were all small, no larger than three cells (Fig. 4F). They were found only among the terminal subclones and within the minority branches of asymmetric subclones (Fig. 4, H to I). This again indicates that ET-PNs do not arise from fated RGPs, but rather from the multipotent population—primarily through indirect neurogenesis involving low-proliferative progenitors that undergo a maximum of two divisions, as indicated by ET-PN subclone sizes (Fig. 4F).

In contrast to ET-PN sublineages, IT-PN sublineages demonstrated progression through RGPs. At E12.5, they showed a wide range of sizes (1 to 15 PNs) (Fig. 4F) and were found more abundantly in asymmetric and terminal divisions (Fig. 4, H and I). Within the asymmetric divisions, the “Majority” branches represent the outputs produced by daughter RGPs that emerge with lineage progression. They, therefore, inform of the decisions taken by the parental multipotent RGP population. On our clones, half of the Majority lineages born from E12.5 asymmetric divisions were Mixed, and the other half were IT-PN sublineages (Fig. 4H). This indicated that after an E12.5 neurogenic division, there is an equal chance for a daughter RGP to maintain multipotency or to become specified for IT-PN production. Our results thus indicate that IT-PN–fated RGPs do not preexist as an independent population but instead arise from half of the neurogenic divisions of multipotent RGPs. Accordingly, by E13.5, IT-PN Majority lineages outnumbered Mixed lineages by two-thirds (Fig. 4H). The magnitude of the increase indicates that the daughter RGPs of IT-PN–fated RGPs inherit the restriction, while the daughter multipotent RGPs behave exactly as the parental multipotent RGPs, expanding the pool of IT-PN–fated RGPs in half of their divisions. In this manner, multipotent RGPs, the source of ET-PNs, are depleted with lineage progression (Fig. 4K).

We then examined the laminar distribution of the outputs produced by IT-PN–fated RGPs (i.e., IT-PN subclones ≥4). Notably, nearly all (90%) of IT-PN sublineages were translaminar; i.e., they produce IT-PNs for both DLs and ULs (Fig. 4J). By E13.5, the fraction of UL-IT-PN subclones increased. Thus, IT-PN–fated RGPs display broad layer potential and produce large translaminar outputs through the sequential generation of DL and UL-IT-PNs.

Overall, our MADM-based clonal analysis demonstrates that the specification of a subset of IT-PN–fated RGPs represents a central decision during neurogenesis. The specification of IT-PN-RGPs is simultaneous to the specification of ET-PN sublineages, which will therefore produce PNs in parallel but mediated by progenitors with limited proliferation capacity. The adoption of such an IT-PN fate by RGPs correlates with a capacity to generate large translaminar clones. With developmental time, the number of these IT-PN–fated RGPs surpasses, population-wise, the pool of multipotent RGPs, in turn ending the production of ET-PNs (Fig. 4K).

### Small IT-PN and ET-PN sublineages from early neurogenic divisions

We next examined the “minority” branches that represent the most immediate neuronal outputs following TM inductions. Notably, at E12.5, most minority lineages contained two to three cells and thus involved indirect neurogenesis. Moreover, most of the minority lineages were pure lineages (Fig. 4H), suggesting that ET-PN or IT-PN fates are not determined in postmitotic PNs, but rather in progenitors—at the very least during the terminal division of their most immediate progenitor. Hence, rather than direct neurogenesis, the multipotent lineages progress through the initial specification of projection-type fated progenitors that give rise to small (self-consuming) IT-PN and ET-PN short sublineages. Somehow unexpected given the EdU birth-dating experiments, we found as many minority IT-PN sublineages as ET-PN sublineages. Such equal occurrence was observed both at E12.5 (48% ET-PNs versus 33% IT-PNs) and at E13.5 (43% ET-PNs versus 57% IT-PNs). The numbers were slightly skewed toward ET-PN lineages at E12.5 and IT-PN lineages at E13.5 (Fig. 4H), but could not account for the earlier production of ET-PNs demonstrated with EdU.

**Fig. 5.**
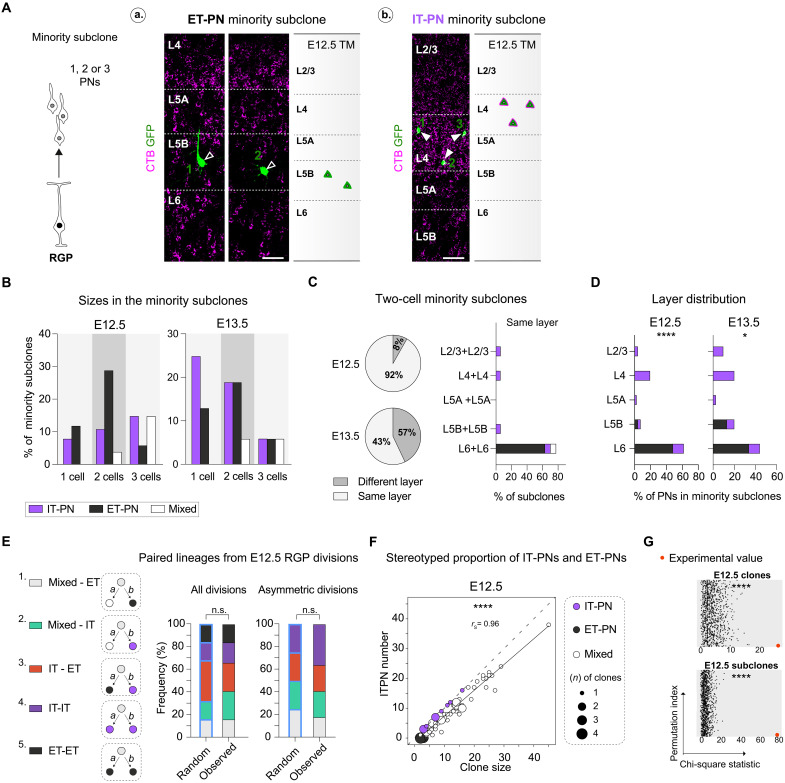
Analysis of IT-PN and ET-PN pure small sublineages. (**A**) (Left) Schematic and (right) representative images and reconstructions of pure ET-PN (a) or IT-PN (b) minority subclones. Empty and white arrowheads indicate CTB^−^PNs and CTB^+^PNs cells, respectively. Scale bars, 50 μm. (**B**) Proportions of pure IT-PN (purple), pure ET-PN (black), and Mixed (white) minority subclones containing one, two, or three PNs labeled during asymmetric E12.5 or E13.5 MADM divisions. (**C**) (Left) Fraction of two-cell minority subclones with neurons in the same layer. (Right) Layer distribution at P10. (**D**) Differences in the layer distribution between IT-PNs and ET-PNs in the minority subclones. Fisher’s test: (E12.5) ****P < 0.0001 and (E13.5) *P = 0.0484. (**E**) (Left) Schematic of the five combinations of paired sublineages and (right) their proportions following TM induction at E12.5. Chi-square test comparing E12.5 distributions to a theoretical random distribution showed nonsignificant differences. (**F**) Plot of IT-PN number versus clone size for clones labeled at E12.5. The bisector (dashed line) indicates IT-PN clones. The black line represents linear regression: *y* = 0.8646*x* clone size – 1.28, where *y* is IT-PN number. Spearman correlation (*r*_s_ = Spearman’s rank correlation coefficient): *r*_s_ (75) = 0.96, ****P < 0.0001. (**G**) Association between clone/subclone type (IT-PN, ET-PN, and Mixed) and the type of division (P/A/T). A chi-square statistic from the observed data was compared to the chi-square statistics from 1000 random permutations of the dataset. The *P* value is calculated as the proportion of permuted chi-square statistics that are greater than or equal to the observed chi-square statistic: ****P < 0.0001.

To investigate the causes of the sequential production of ET-PNs and IT-PNs demonstrated with EdU, we classified the minority outputs in one-cell, two-cell, and three-cell minority lineages (Fig. 5, A and B). IT-PN production showed any one-to-three clone size while ET-PN lineages were enriched in two-cell subclones (Fig. 5B). This suggests distinct modes of proliferation within the sublineages. Besides, ET-PN minority subclones were often PN pairs of the same layer, indicating symmetric terminal divisions (Fig. 5C). Together, these results strongly suggest that ET-PNs are preferentially generated via IPs derived from multipotent RGPs, which, according to EdU results, undergo terminal divisions around E13.5. In contrast, more than one-third of the IT-PNs produced in the minority subclones were found in the ULs (Fig. 5D). Because UL-PNs are born at E14.5 onward, this result indicates that minority IT-PN subclones involve progenitors that produce two to three PN outputs over a prolonged time window (E12.5 to E14.5) and progress differently from ET-PN sublineages. Reported progenitors with such behavior include self-consuming RGPs and other types of intermediate low-proliferative neuronal progenitors (*43*–*45*).

Our MADM-based clonal analysis supports the idea that small ET-PN and IT-PN subclones involve progenitors that produce outputs with distinctive modes of progression. While elucidating the precise types of progenitors involved in each sublineage will require further investigation, this result provides a mechanistic explanation for the temporal offset in ET-PN and IT-PN birthdates. In sum, the analysis of small sublineages demonstrated that the principal neurogenic output of multipotent RGPs are pure, small, self-consuming lineages of either ET-PNs or IT-PNs. The divergent progression of the two types of small lineages accounts for the temporal offset between ET-PN and IT-PN birthdates.

### IT-PN–fated RGPs orchestrate a stereotyped neurogenesis

Our data aligned with previous studies indicating that an initial population of multipotent RGPs gives rise to all PNs in the cortex (*17*). However, we found that E12.5 RGPs produce ET-PN and IT-PN lineages simultaneously, not sequentially. Their frequencies also challenge the idea of a unique preprogrammed sequence of stereotyped neurogenic divisions.

To evaluate the nature of fate decisions in the multipotent RGPs, we compared how the sibling sublineages pair with each other. Theoretically, independent decisions in the two daughter cells would produce all possible combinations of paired sublineages in random frequencies (Fig. 5E). Conversely, if a multipotent RGP generated ET-PN and IT-PN lineages in a stereotyped manner, IT-PN lineages would never pair with multipotent (Mixed) RGPs. Instead, ET/Mixed and ET/IT pairs should be the predominant combinations.

In E12.5 MADM divisions, i.e., the multipotent population, we found all possible combinations of paired sublineages in frequencies that support random production in the total clones. In the neurogenic asymmetric clones, the distributions appeared compatible with random outcomes, although the lower number of clones limited statistical power (Fig. 5E) (Materials and Methods). Hence, the frequencies of types of divisions we observed entail the possibility that the activation of an IT-PN restriction after the neurogenic division of a multipotent RGP occurs randomly and independently of the fate of the small sublineages produced in the same division. As a result, multipotent RGPs would progress through a variety of neurogenic sequences that differ at the initial divisions but are stereotyped once an IT-PN–fated RGP emerges. In this scenario, the small and equal frequencies of E12.5 IT-PN and ET-PN clones that we report could be simply the statistical chance result of two sibling cells activating independently the same projection-type identity program.

Next, to evaluate whether the observed frequencies of clones render an overall quantitative stereotyped process at population level, we plotted clone size (*n*) against the number of IT-PNs. This analysis showed a broad range of sizes for Mixed clones (white dots) in both E12.5 and E13.5 populations, with the former being generally larger. Notably, linear regression analysis revealed a robust relationship between IT-PN content and clone size and demonstrated, on average, a proportional parallel increase in IT-PNs and ET-PNs with clone size (Fig. 5F and fig. S7). This indicated that the observed production modes render a predictable population, whereby the larger clones are composed of smaller, repeated modular units. Moreover, nine PNs was the most frequent unit size of asymmetric clones (fig. S6E) in agreement with earlier MADM analyses (*8*). Thus, per our linear regression, a canonical E12.5 neurogenic RGP produces an average of six to seven IT-PNs and two to three ET-PNs (Fig. 5F). Last, probabilistic analysis demonstrated a strong correlation between clone/subclone size and projection type (ET-PN, IT-PN, or Mixed) (Fig. 5G). This indicated that multipotent, ET-PN, and IT-PN lineages and sublineages each correlate with specific sizes, supporting that proliferation and PN specification are intrinsically linked. Overall, our MADM-based analysis reveals that, although the neurogenic process may not follow a unique stereotyped initial division, it yields a quantitatively and temporally stereotyped PN production.

### POU3F transcription factors drive IT-PN fate specification

**Fig. 6.**
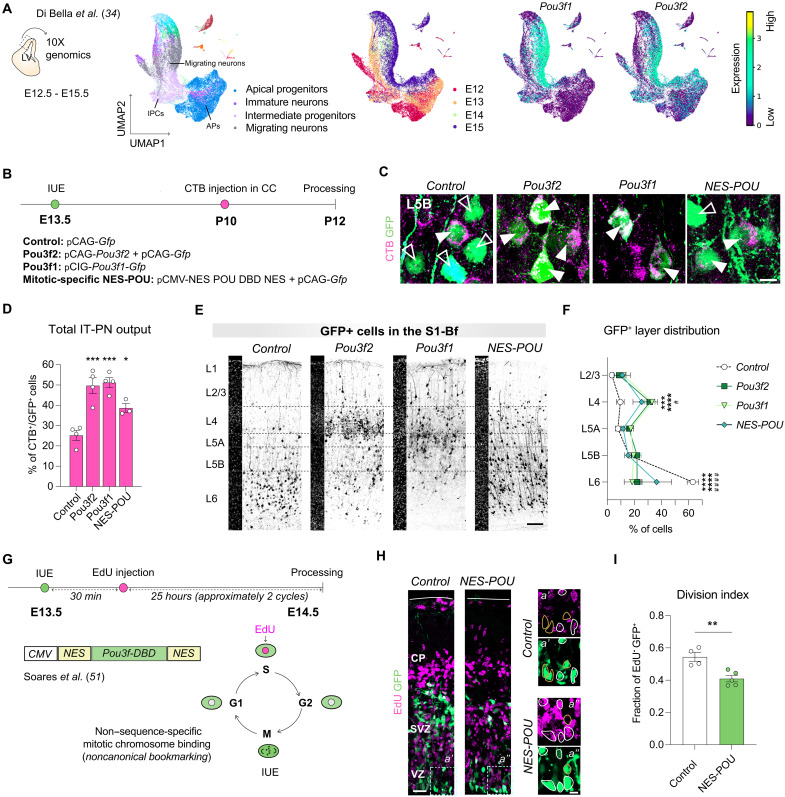
POU3F proteins contribute to IT-PN specification. (**A**) (Left) UMAP plots showing annotated cell populations across developmental stages (E12.5 to E15.5) in the Di Bella *et al.* (*34*) dataset. (Right) Expression of *Pou3f1 and Pou3f2* plotted in the UMAP. (**B**) Experimental design. (**C**) Images of electroporated neurons in L5B. GFP in green and CTB in magenta. Empty and filled white arrow indicated CTB^−^ and CTB^+^ labeling, respectively. (**D**) Percentage of IT-PNs over the electroporated population. Percentages were calculated from the total number of GFP^+^ cells in the S1-Bf area. Mean ± SEM (*n* = 2 sections per animal; *n* ≥ 3 animals per condition). One-way ANOVA with Dunnett’s post hoc test: ***P_Control versus Pou3f2_ = 0.0003, ***P_Control versus Pou3f1_ = 0.0002, and *P_Control versus NES-POU_ = 0.0286. (**E**) GFP signal (black) in the S1-Bf at P12 following IUE at E13.5 with the corresponding plasmids. (**F**) Layer distribution of electroporated PNs. Mean ± SEM (*n* = 2 sections per animal; *n* ≥ 3 animals per condition). Two-way ANOVA with Tukey’s post hoc test: ***P_L4 Control versus L4 Pou3f2_ = 0.005, ****P_L4 Control versus L4 Pou3f1_ < 0.0001, ^#^*P*_L4 Control versus L4 NES-POU_ = 0.0311, ****P_L6 Control versus L6 Pou3f1_ < 0.0001, ****P_L6 Control versus L6 Pou3f2_ < 0.0001, and ^####^*P*_L6 Control versus L6 NES-POU_ < 0.0001. (**G**) Experimental design (*51*). (**H**) Representative images of the electroporated embryos. Right panels show high magnification of the area indicated with white boxes (left panel). White-shaped: EdU^+^GFP^+^ cells; yellow-shaped: EdU^−^GFP^+^ cells. (**I**) Division index expressed as the fraction of EdU^−^GFP^+^/total number GFP^+^ cells. Mean ± SEM (*n* ≥ 3 animals per condition). Unpaired *t* test: **P= 0.0045. Scale bars, 10 μm (C), 100 μm (E), 25 μm [(H) low magnification], and 10 μm [(H) high magnification].

MADM clonal analysis revealed that E12.5 RGPs give rise to IT-PN–fated RGPs. To identify molecular determinants underlying an IT-PN fate restriction in RGPs, we analyzed a previously published single-cell RNA sequencing dataset of cortical cells from E12.5 to E15.5 (*34*). In the study, progenitors were annotated as APs and IPs, the former containing RGPs. Notably, UMAP embedding of cells clustered by cell type and developmental stage (Fig. 6A and fig. S8, A to C) revealed an interesting expression profile for *Pou3f* transcripts. POU3F TFs are known to influence UL-PN numbers and identity (*46*–*49*) and to regulate proliferation, cell cycle exit, and the balance between direct and indirect neurogenesis in murine cortical progenitors (*50*). *Pou3f1*/*2*/*3* RNAs showed undetectable levels in early-born PNs, but from E14.5 onward, they were found in clusters of migrating and immature neurons. In progenitors, most IPs from E14.5 to E15.5 cortices expressed *Pou3f1/2/3*, whereas these transcripts were detected in only a fraction of IPs from E12.5 to E13.5 cortices. Most notably, in APs, *Pou3f2* identified increasingly larger subsets of cells from E12.5 to E15.5, *Pou3f3* showed intermediate levels of expression in all APs invariably across all stages, and *Pou3f1* levels remained low at all stages (Fig. 6A and figs. S8, C and D, and S9A). Hence, *Pou3f* genes, and most particularly *Pou3f2* expression, showed temporal changes expected from an IT-PN fate in RGPs.

To investigate whether POU programs regulate IT-PN production, we performed IUE to overexpress *Pou3f2* in E13.5 VZ progenitors (Fig. 6, B to F). We also tested the effects of *Pou3f1*, which shows the highest DNA affinity among all POU TFs, and its overexpression ensures a broad efficient modulation of POU programs (*46*). The plasmids included *Gfp* sequences for the visualization of electroporated neurons. To identify electroporated IT-PNs, we injected CTB into the CC at P10 and quantified the proportion of CTB^+^GFP^+^ cells at P12 (Fig. 6B).

In the control condition, most of the E13.5 GFP^+^ outputs were composed of CTB-negative PNs (ET-PNs) that migrate to the DLs (Fig. 6, C to F). Overexpression of *Pou3f2* or *Pou3f1* increased the proportion of CTB^+^GFP^+^ cells (IT-PNs) from 20% to approximately 50% of the GFP^+^ population (Fig. 6D) and shifted their position toward the ULs (Fig. 6, E and F). These data indicate that POU programs contribute to IT-PN specification.

In addition, we similarly examined loss-of-function conditions (fig. S9, B to G). Knocking down *Pou3f2* alone or both *Pou3f1* and *Pou3f2* by electroporation of E13.5 progenitors showed no significant effects in IT-PN content or migration (fig. S9, E to G). However, combined *Pou3f1/2* knockdown resulted in a broadened GFP^+^ PN distribution across layers (fig. S9, F and G) and shifted the CTIP2^+^ (ET-PNs):CTB^+^ (DL-IT-PNs) ratio to 1:1, compared to 3:1 in controls (fig. S9, H and I). These alterations supported that in the absence of *Pou3f*, multipotent RGPs produce only small sublineages and not the IT-PN–fated RGPs that would continue to proliferate and dilute the GFP plasmids.

Next, because the initial POU3F1/2 overexpression experiments could not separate effects on RGPs and their PN outputs, we investigated the function of POU3F proteins specifically in dividing progenitors using a previously engineered *Pou3f2* construct (NES-*Pou3f*). NES-*Pou3f* contains the POU-specific and POU-homeodomain DNA-binding domains of POU3F2 fused to a nuclear export signal (NES) and lacks both N- and C-terminal transactivation domains (Fig. 6B). This design permits chromatin-binding specifically at mitosis, when the nuclear membrane is disassembled, but prevents transcriptional activation (*51*). Using this construct, it has been demonstrated that POU3F proteins bind to mitotic chromatin in neural stem cells. Notably, this binding does not involve the recognition of POU binding sites in the chromatin, but is mediated by sequence-independent electrostatic interactions with the DNA. This noncanonical mitotic bookmarking regulates the reactivation of a large cluster of genes after the division of neural stem cells, including *Nestin* and *Fabp7* (*51*), genes also expressed in RGPs (*52*, *53*).

We overexpressed NES-*Pou3f* in E13.5 ventricular progenitors and analyzed the content of IT-PNs in the outputs. NES-*Pou3f* overexpression led to an increased production of IT-PNs, resembling the partial effect observed upon overexpression of wild-type POU3F proteins (Fig. 6D). Consistent with this, we observed a shift in the laminar position of the neuronal output toward layer 4 (Fig. 6, E and F). These results indicate that overexpression of POU3F proteins biases PN fate output by acting on cortical progenitors. The data support a model in which noncanonical mitotic bookmarking by POU3F proteins may contribute to IT-PN specification during mitosis.

As with conditions overexpressing POU3F WT forms, NES-*Pou3f* resulted in migration to more superficial cortical layers, all mirroring observations in the IT-PN sublineages in the MADM analysis and suggesting changes in cell division associated to IT-PN production. To assess this possibility, we administered a pulse of EdU to pregnant dams 30 min after electroporating the NES-*Pou3f* plasmid into E13.5 progenitors and analyzed nucleotide dilution 26 hours later—a time window encompassing up to two rounds of mitosis (Fig. 6G). Compared to controls, cortical progenitors overexpressing NES-*Pou3f* showed a smaller fraction of EdU^−^GFP^+^ cells (Fig. 6, H and I), indicating lower rates of EdU dilution. This finding is consistent with a lengthened cell cycle or a shift from indirect to direct neurogenesis.

Altogether, our findings support a modulatory or even instructive role for POU3F transcription factors in biasing IT-PN fate, potentially involving non–sequence-specific mitotic chromosome bookmarking. Furthermore, they suggest that this fate shift is coupled with changes in the proliferative behavior and temporal dynamics of RGPs during cortical development.

## DISCUSSION

**Fig. 7.**
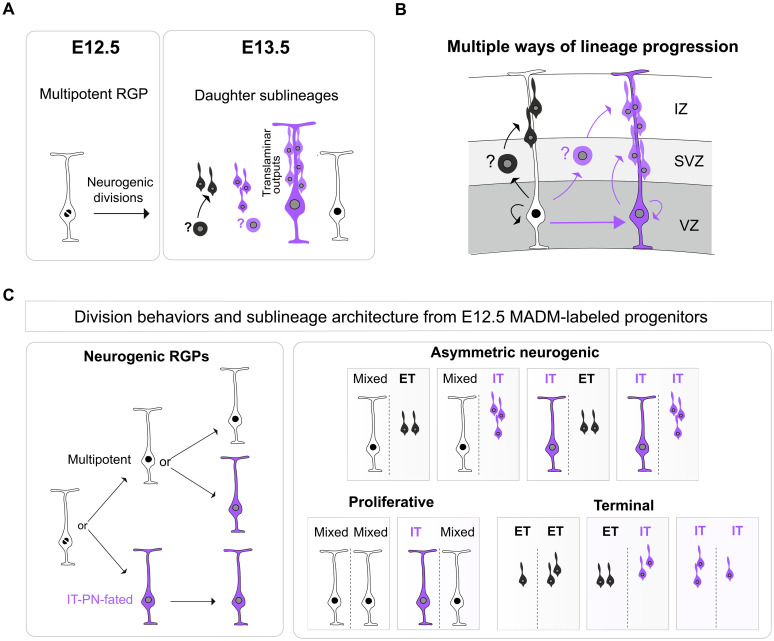
Model of the lineage progression of multipotent RGPs. IT-PN sublineages are depicted in purple, ET-PN sublineages are shown in black, and multipotent RGPs are represented in white. (**A**) Neurogenesis progresses through fated sublineages. Upon entering neurogenesis at E12.5, four types of subclones emerge from the multipotent RGP population: small self-consuming ET-PN (most frequent size 2), small IT-PN lineages (most frequent size 3), large translaminar RGP-mediated IT-PN subclones, and multipotent subclones (Mixed, comprising ET-PN and IT-PN). (**B**) The distinct combinations of daughter sublineages reveal multiple ways of lineage progression. These trajectories differ only at the early divisions, and once the IT-PN–fated RGP is specified, the lineages follow the same progression. (**C**) Division behaviors and sublineage combinations traced in this study with MADM clonal analysis. (Left) In the canonical asymmetric divisions generating a Majority and a minority subclone, we observe that neurogenic multipotent RGPs divide and generate RGP daughter progenitors that with equal probability (i) are IT-PN–fated RGPs or (ii) maintain multipotency. (Right) Scheme illustrating the types of sublineage combinations observed in this study with MADM clonal analysis. The frequencies of the combinations do not allow the possibility that the specification decision in the Major and minor subclones could be independent events.

The data in our study support a model in which projection-type specification is linked to distinct sublineages and constitutes an integral dimension of cortical lineage progression. By incorporating an early ET/IT bifurcation into the classical laminar schema, this study refines the current framework for RGP lineage progression and provides a more comprehensive view of how RGPs generate PN diversity in the developing neocortex. While consistent with previous research indicating that all mouse cortical PNs originate from an initially homogeneous population of multipotent RGPs (*8*, *10*, *19*, *54*), our clonal analysis provides evidence that early projection-identity decisions can shape distinct cortical lineage behaviors, complementing the classical inside-out model of cortical development (Fig. 7). Thus, rather than being dictated solely by sequential laminar progression, a broad projection-type identity appears specified in parallel with laminar fates across two sublineages. This suggests that projection-type specification constitutes an additional organizational axis that operates in parallel with the canonical inside-out sequence during RGP lineage progression.

The early neurogenic bifurcation that segregates ET and IT lineages seems coupled to distinct modes of sublineage progression. ET-PN progenitors are low-proliferative and produce small clones restricted to the DLs. In contrast, IT-PN progenitors can produce large, translaminar lineages that include PNs across both DLs and ULs. The complementary analysis of E12.5 and E13.5 MADM-labeled clones supports the idea that IT-PN lineages arise from and progress through RGPs, enabling their characteristic translaminar output. We thus refer to this RGPs as IT-PN–fated RGPs, indicating a reduction of the initial multipotent potential. The identification of IT-PN–fated RGPs early in lineage progression is a key finding of this study.

Another important aspect of our data is the mode of lineage decisions, which we examined to determine whether they follow deterministic or stochastic patterns. At the population level—and despite the apparent complexity of individual lineage trajectories—RGP production of PNs is highly stereotyped. A major contributor to this orderly output appears to be the emergence of IT-PN–fated RGPs, which impose constraints that enforce a temporally and quantitatively ordered pattern of PN production across the progenitor pool.

With regard to the initial mode of division of multipotent RGPs, our data do not allow strong conclusions. While our statistical analysis does not support a preprogrammed mechanism acting at the level of individual progenitors, the number of neurogenic E12.5 divisions captured experimentally is also not sufficient to rule this possibility out. Nevertheless, the observed frequencies of sublineages derived from E12.5 divisions are compatible with the hypothesis that the first neurogenic decision is inherently noisy. Such a mechanism would provide a parsimonious explanation for how large-scale reproducibility in cortical neuron numbers can emerge from diverse lineage behaviors. This interpretation aligns with previous MADM studies showing that not all clones contained PNs from all layers (L2 to L6) but instead exhibited variable layer configurations (*8*, *15*, *18*). It also aligns with higher stochasticity during early RGP divisions, followed by progressively increasing fate restrictions that narrow RGP lineage trajectories later in development (*28*, *55*, *56*). Hence, the early bifurcation we observe may reflect stochastic influences, although within the constraints of our study, a deterministic mechanism cannot be conclusively excluded.

Another key insight from our analysis is that projection identities are specified within parallel lineages, each following an intrinsic inside-out sequence of laminar generation. Nevertheless, these lineages give rise to PNs that ultimately settle in the same cortical layer despite being produced at distinct developmental time points. This organization suggests that laminar position and projection identity, though coordinated, can be uncoupled across lineage branches, revealing a more nuanced temporal and lineage-based logic underlying PN subtype specification. This departure from the classic inside-out migration pattern was already hinted at in early birth-dating studies of the mouse cortex (*26*). More recent work has shown that isochronically generated neurons can adopt diverse laminar identities (*28*). Our results extend this emerging view by suggesting that an early, broad projection-subtype decision is made over sequential laminar identities through distinct sublineages. The pattern of output production within the ET-PN and IT-PN lineages appears different, which may play a role in establishing distinct laminar identities, although the precise nature and extent of these differences remain to be fully defined. At the same time, evidence from mouse models further supports the idea that PN subtype identity can be maintained independently of final laminar position. In *Reeler* mutants, long-range connectivity remains largely preserved despite severe disruptions in cortical layering (*57*). Similarly, in *Eml1* knockouts, heterotopic neurons retain their molecular identity and appropriate projection patterns (*58*). These findings raise a central question: how and when is laminar fate resolved—during migration, through lineage-intrinsic programs, or via interactions between intrinsic and extrinsic cues? (*59*).

The results support the emerging view that progenitors can carry projection-subtype identity, as proposed in recent studies (*16*, *27*, *34*). In our study, the small sizes and the high frequency of PN pairs observed in ET-PN lineages resemble a canonical IP progenitor division, a possibility that could be tested in the future through targeted genetic manipulation of IPs (*29*). For IT-PN lineages, our data indicate a shift in the mode of RGP division, with indirect neurogenesis predominating at E12.5 and more direct neurogenesis at E13.5—an effect to which POU3F transcription factors may contribute (*50*). Notably, slower IT-PN production rates are underscored by our observation of small IT-PN subclones in which L4 PNs constitute the earliest progeny of an E12.5-derived progenitor. This pattern aligns with several progenitor populations previously implicated in UL-PN neurogenesis, including slow-cycling RGPs associated with UL production (*14*, *60*); RGP-derived apical IPs, or short neural precursors (SNPs), which exhibit prolonged cell-cycle kinetics and preferentially generate L4 PNs (*43*–*45*); and outer SVZ RGPs (oRGs/bRGs), a progenitor class strongly linked to IT-PN generation in humans and nonhuman primates (*61*, *62*). A further possibility involves IPs that undergo multiple rounds of division, although such events appear relatively rare in mice (*63*, *64*).

In our study, MADM-based lineage reconstruction enables the retrospective inference of an early projection-type bifurcation and its progression through parallel sublineages. However, direct evidence for an RGP specification is still lacking. Mechanistically, our findings provide evidence for a modulatory or instructive role of POU-domain mitotic binding in IT-PN fate bias. POU3F2 and POU3F3 regulate proliferation and cell-cycle exit in mouse RGPs, and their loss of function reportedly impairs UL-PN production (*46*, *50*). Furthermore, *Pou3f2* transcript dynamics support the idea that PN diversity arises from early transcriptional heterogeneity within RGPs. Herein, we show that a truncated POU3F protein containing only the POU-specific and POU-homeo DNA-binding domains, together with a NES, is sufficient to induce IT-PN production from E13.5 VZ progenitors. This mutant binds chromatin only during mitosis and mediates a noncanonical bookmarking mechanism in which POU domains interact with chromatin in a non–sequence-specific manner (*51*). Such bookmarking may preserve a permissive chromatin state or modulate chromatin architecture to favor IT-PN identities (*65*, *66*). This mechanism may also have disease relevance. POU3F2 variants are associated with bipolar disorder and schizophrenia (*67*), and *Pou3f2* loss-of-function mutations in mice cause microcephaly (*50*), a phenotype that may reflect impaired IT-PN specification. IT-PNs represent the most expanded and diversified PN class across mammals (*61*, *68*) and are particularly vulnerable in neuropsychiatric disorders (*67*, *69*). Conversely, it is tempting to speculate that the emergence and enrichment in IT-PN–fated lineages, which proliferate as RGPs, enabled increased PN output and contributed to mammalian cortical expansion.

Last, a few methodological aspects deserve more detailed discussion. Birth-dating analyses were based on single-pulse EdU labeling. Although EdU birth-dating has inherent limitations, the temporal offset in the production of IT and ET populations in the DLs was well resolved in double-injected animals—ruling out artifacts due to developmental variability among pups. These findings were further validated by IUE. Complementary approaches such as FlashTag (*70*), barcoding (*71*), or MADM Clone-seq (*72*) would further integrate temporal, positional, and molecular information at clonal resolution.

On the other hand, we were careful to base our classification of clones on the positive identification of IT-PNs labeled from the CC. Our retrograde tracing paradigms did not capture some E12.5 neurons (potentially, TLE4^+^ neurons). Because these outputs are not retrogradely labeled from the CC, they are appropriately classified as ET-PNs in the MADM analysis. This rationale underlies our decision to use CC injections to assess IT-PN content rather than ET-PN injections when analyzing MADM outputs. Nevertheless, it should be noted that our results might underestimate the heterogeneity in the ET-PN lineages. This unobserved complexity could mask a pattern of fate decisions not resolved by our clonal analysis, potentially shifting the interpretation of progenitor behavior away from a purely random model.

In addition, because MADM lines are maintained on a mixed genetic background, it should be noted that our lineage conclusions are specific to the MADM genetic context. Nevertheless, control experiments revealed no detectable differences in PN production timing relative to inbred C57BL/6 mice (fig. S6J).

In summary, our analysis of RGP lineage progression and early neurogenic divisions refines and extends the classical view of multipotent RGPs progressing through a linear, unique neurogenic sequence. Our data support an RGP lineage progression model that includes the early emergence of parallel fated sublineages. The specification of IT-PN–fated daughter RGPs, associated to a shift to direct neurogenesis, ensures the expansion of the IT-PN lineage and a globally stereotyped neurogenic process. These findings introduce innovative perspectives of RGP lineage architecture in the mammalian neocortex.

## MATERIALS AND METHODS

### Animals

Wild-type *C57BL6/J* mice (the Jackson Laboratory JAX no. 000664) were used for all experiments except for MADM. For MADM analysis, we used previously described transgenic mouse lines with MADM cassettes inserted in chromosome 11 (*42*): *MADM-11-GT* (*MADM^GT/GT^*; the Jackson Laboratory JAX no. 013749) and *MADM-11-TG* (*MADM^TG/TG^*; the Jackson Laboratory JAX no. 013751). We crossed *MADM-11-GT* mice with the driver line *Emx1*-*CreERT*^2^ (*73*) for specific cortical expression. These mouse lines were kept in mixed C57/Bl6, FVB, and CD1 genetic background.

Males and females of WT or the desired genotypes were used randomly for all experiments. For pregnant dams, the morning of the appearance of a vaginal plug was defined as embryonic day (E) 0.5. Animals were housed at 21° ± 1°C ambient temperature and 40 to 55% humidity under a 12:12-hour light/dark cycle. Water and food were provided ad libitum. Animals were bred and maintained following the guidelines from the European Union Council Directive (86/609/ European Economic Community). All procedures involving the handling and euthanasia of animals complied with European Commission guidelines (2010/63/EU). All animal procedures were approved by the CSIC, the Community of Madrid Ethics Committees on Animal Experimentation, and by the Austrian Federal Ministry of Science and Research, in compliance with national and European legislation (PROEX 123-17; 124-17; 158.7/21; 230.7/21; 159.5/22; BMWFW-66.018/0006-WF/V/3b/2017 and BMWFW-66.018/0015-WF/V/3b/2017).

### CTB injections for retrograde labeling

Axonal retrograde labeling was performed by injecting CTB conjugated to Alexa Fluor 488, 555, or 647 (Thermo Fisher Scientific, catalog nos. C-34775, C-34776, and C-34778) into the CC, the IC, the CerbPed, or the dorsal striatum. Stereotaxic coordinates (in millimeters) and CTB volumes are reported in Tables 1 and 2. For CTB injections performed between P0 and P5, to ensure the completion of neuronal migration and NeuN expression, we euthanized the animals at P10. For other injections, animals were euthanized 2 days post-CTB injections to allow for retrograde transport of the tracer (*33*, *35*). In MADM experiments, two injections were performed into the CC using different coordinates along the anteroposterior axis to cover the entire tract width in the SS cortex. Anesthesia during the entire surgical procedure was given using isoflurane/oxygen (3% isofluorane for induction, 1.5 to 2% isofluorane for maintenance during the surgery). Anesthetized animals were placed on a stereotaxic frame (Harvard Apparatus) with a mouse neonatal adapter (Stoelting). CTB, diluted at 0.5% in phosphate-buffered saline (PBS), was injected with a Drummond Nanoject II Auto-Nanoliter Injector using 30-mm pulled glass micropipettes (Drummond Scientific, ref. 3000205 A and 3000203 G/X). CTB was delivered at a rate of 9.2 nl per injection pulse with a maximum frequency of one pulse per second (9.2 nl/s) to minimize damage until the desired total volume was achieved. Animals were perfused for analysis at least 48 hours postinjection.

**Table 1.** CTB coordinates and volumes for CC injections.

Injection site Anatomical reference point (0,0,0) bregma	Postnatal day (P)	Injected volume (nl)	Angle	Coordinates (mm)		
				AP (anteroposterior)	ML (mediolateral)	DV (dorsoventral) pulses at 0.05 mm
Somatosensory CC	P1/P0	230	0	−1	+0.5	−0.8 to −1.1
	P2	230	0	−1	+0.5	−0.9 to −1.2
	P3	322	0	−1	+0.5	−0.95 to −1.25
	P4	322	0	−1	+0.5	−1.05 to −1.35
	P5 (anterior)	460	0	−1	+0.5	−1.05 to −1.35
	P5 (posterior)	460	0	−1.4	+0.5	−1.05 to −1.35
	P10	460	18	−1.4	+0.7	−1.3 to −1.6
	P16	575	18	−1.4	+0.7	−1.6 to −1.9
	P30	575	18	−1.4	+0.7	−1.7 to −2

**Table 2.** CTB coordinates and volumes for ET injections.

Injection site Anatomical reference point (0,0,0) bregma	Postnatal day (P)	Injected volume (nl)	Angle	Coordinates (mm)		
				AP (anteroposterior)	ML (mediolateral)	DV (dorsoventral)
Dorsal Str	P5	27.6	20	−1.4	+3.5	−2.3
IC	P5	27.6	10	−1.4	+2.7	−2.7
	P16	27.6	10	−1.4	+2.7	−3
CerbPed	P5	27.6	0	+0.8	+2	−3.8
	P16	27.6	0	+0.8	+2	−4

### EdU birth-dating

Pregnant dams were intraperitoneally injected with the thymidine analog, EdU (Merck, catalog no. 900584) (50 mg/kg body weight), using a 30G needle at different stages of embryonic development (E11.5, E12.5, E13.5, E14.5, or E15.5). The pups were transcardially perfused at P10. Brains were collected, sectioned, and processed for the detection of EdU using the Click-It Alexa Fluor imaging kit according to the manufacturer’s instructions (Thermo Fisher Scientific, catalog nos. C-10337 and C-10340).

### MADM genetic clonal labeling

*MADM-11^GT/GT^; Emx1-CreERT^2+/−^* mice were crossed with *MADM-11^TG/TG^* mice using males and females of both strains indistinguishably. MADM clone induction was performed as previously described (*8*, *74*). Timed pregnant dams were injected intraperitoneally at E12.5 or E13.5 with tamoxifen (TM) (Sigma-Aldrich, catalog no. T5648-5G) dissolved in corn oil (Sigma-Aldrich, catalog no. C8267) at a dose of 2 mg per pregnant dam from a stock prepared at 20 mg/ml. Embryos were recovered at E18 to E19 through cesarean section, fostered and raised for CTB injection at P5, and euthanized at P10 for analysis.

MADM-based clonal analysis with *MADM-11* transgenic lines has been previously validated to identify clusters of cells, each representing individual clonal units (*9*, *42*, *74*). The TM dose used in this study has been proven to achieve sparse labeling (*8*). A 300-mm minimum distance was used to determine separate clones in brains with multiple cell clusters.

### MADM taxonomy

To identify MADM clones, we first analyzed GFP^+^ and tdTomato^+^ cell clusters in each section sequentially, following a rostro-caudal order. High-magnification images of each individual neuron were acquired for assessing CTB labeling. Each clone was individually annotated for the presence or absence of CTB labeling and layer occupancy in an atlas (see Clone Atlas in the Supplementary Materials). For MADM taxonomy, we positively assigned IT-PN identity while we applied a negative classification for ET-PNs. Clones and subclones were categorized into three classes: IT-PN, consisting exclusively of IT-PNs (i.e., UL-PNs and CTB^+^ DL-PNs); ET-PN, consisting exclusively of CTB^−^ DL-PNs; and Mixed, containing both IT-PNs and ET-PNs. As a control, to ensure the correct discrimination of DL-IT-PNs from ET-PNs in MADM experiments, we verified that CTB labeling efficiencies in MADM^+^ PNs were comparable to the values we obtained in WT mice in NeuN^+^ cells. Control experiments confirmed the CTB labeling efficiency of MADM outputs comparable to WT animals, and showed cell death rates and UL/DL-PN ratios (fig. S6) consistent with previous reports (*8*).

### IUE and plasmids

IUE was performed as previously described (*75*). The uterine horns of anesthetized pregnant females were extracted following a longitudinal incision in the epithelial tissue and laparotomy in the muscle. Plasmid DNA at 1 μg/μl: pCIG2-*Pou3f1* (gift from M. Cayouette), pCAG-*Pou3f2* (Addgene, plasmid no. 19711), and *CMV-NES-Pou3f2DBD-NES* (*51*), or plasmid DNA at 0.8 μg/μl: pLKO.1-*shPou3f1* and pLKO.1-*shPou3f2* (Sigma-Aldrich). In all conditions, pCAG-*Gfp* (1 μg/μl; Addgene, plasmid no. 11150) plasmid was co-electroporated to visualize electroporated cells. Plasmid DNA solutions were prepared with 0.1% Fast Green in ddH2O (Sigma-Aldrich, catalog no. F7252). The plasmid solutions were injected in the lateral ventricle of the embryo using a 30-mm pulled glass micropipette. Five pulses of 50 ms duration were applied at voltages of 33 V (E13.5), 36 V (E14.5), and 38 V (E15.5) using external paddle electrodes (Sonidel Limited, ref. CUY650P5) oriented to target the primary somatosensory (S1) cortex. Electroporation was performed with an ECM 830 electroporator (BTX, ref. 650052). Afterward, the uterine horns were introduced inside the abdominal cavity, and the muscle incision was sutured. An intraperitoneal injection of carprofen solution (Rimadyl, Zoetis, catalog no. 10001515) was applied to prevent postoperative pain. For E13.5, a cesarean section at E19.5 was performed if required. Pups were allowed to develop normally until the stage of interest. Immunostaining against GFP was performed as described below for signal amplification.

### In vitro validation of short hairpin RNAs

To validate the efficacy of the short hairpin RNAs (shRNAs) in vitro, N2A cells were cultured and transfected with the respective plasmids using Effectene (Qiagen, catalog no. 301425). Four shRNAs targeting *Pou3f2* and five shRNAs against *Pou3f1* were tested, alongside a scrambled shRNA control. shRNAs were co-transfected with the overexpression plasmids (*pCAG-Pou3f2* and *pCAG-GFP*, or *pCIG-Pou3f1*) (fig. S9, B and C). Transfection efficiency was monitored using GFP as a reporter. After 72 hours, total RNA was extracted from the cells using the RNAspin Mini Kit (Merk, GE Healthcare, catalog no. GE25-0500-72). RNA purity and concentration were verified using a NanoDrop spectrophotometer. cDNA synthesis was performed with 2 μg of RNA using M-MLV reverse transcriptase (Promega, catalog no. M1701) according to the manufacturer’s protocol. Real-time PCR was conducted in a 10-μl reaction volume using SYBR Green Master Mix (Thermo Fisher Scientific, catalog no. A46012). The QuantStudio 3 Detection system (Applied Biosystems) was used under standard Applied Biosystems cycling parameters (40 cycles: 95°C for 15 s, 60°C for 45 s). Primer sequences were designed with Benchling (https://benchling.com/), and cDNA dilutions were optimized to match the amplification efficiencies of housekeeping genes (18S rRNA and Rpl13A). Relative mRNA expression (fig. S9C) was calculated following the ΔCt method, using the Best Keeper index (*76*) as a reference for the total mRNA load in the sample.

### Perfusion and tissue collection

Mice were deeply anesthetized by an intraperitoneal injection of a Xylazine (Xilagesic, Calier) and Ketamine (Imalgene Merial Laboratorios) solution. Animals were transcardially perfused with PBS and either ice-cold 10% formalin solution (Sigma-Aldrich, catalog no. HT501128-4L) or 4% paraformaldehyde (Sigma-Aldrich, catalog no. 441244-1KG) in PBS for MADM experiments. Brains were dissected and postfixed in the fixative for 4 to 12 hours. Cryoprotection was achieved by immersion in 30% sucrose solution in PBS (Merck, catalog no. S0389) for 48 hours. Then, brains were embedded in Tissue-Tek Optimal Cutting Temperature (O.C.T.) (Sakura Tissue-Tek, catalog no. 4583) and stored at −20°C until further use. Tissue was sectioned with a cryostat in coronal orientation at 50 μm thickness, collected in PBS, stained when required, and mounted onto glass slides. Brain cryosections containing MADM clones were collected and kept in the same left-right orientation and in serial order, in multiple 24-well plates containing PBS.

### Brain slice processing, immunostaining, and antibodies

Free-floating sections were used for immunostaining procedures. Sections were immunostained with antibodies overnight at 4°C or room temperature in a solution composed of PBS with 0.5% Triton (PBS-T) (Sigma-Aldrich, Triton X-100) and 5% fetal bovine serum (FBS; Thermo Fisher Scientific, catalog no. A5256701). After washing with PBS, slices were incubated with the secondary antibodies diluted in 2% FBS in PBS-T. Cell nuclei were stained by 15 min incubation with PBS containing 4′,6-diamidino-2-phenylindole (DAPI; 1:1000 dilution, Thermo Fisher Scientific, catalog no. D1306). Sections were embedded in aqua polymount (Aqua-Poly/mount, Polysciences Inc., catalog no. 18606) and stored at 4°C until image acquisition. For MADM analysis, brain sections were mounted on Superfrost glass slides (Thermo Fisher Scientific, catalog no. 22-037-246) and embedded in mounting medium containing Mowiol 4-88 (Carl Roth, catalog no. Art.-No. 0713) and 1,4-diazabicyclooctane (DABCO; Carl Roth, catalog no. 0718). For embryonic tissue, brain sections were mounted on Polysine Adhesion slides (Thermo Fisher Scientific, catalog no. J2800AMNZ). For antigen retrieval, sections were immersed in a citrate solution (Sigma-Aldrich, catalog no. C9999) and heated to 110° for 1 min in a decloaking chamber (Biocare Medical, catalog no. DC2012).

The following primary antibodies were used: mouse monoclonal anti-NeuN (1:500, Merck Millipore, catalog no. AB377/A60), rabbit polyclonal anti-GFP (1:500, Thermo Fisher Scientific, catalog no. A-11122), mouse monoclonal anti-TBR2 (1:100, eBioscience–Thermo Fisher Scientific, catalog no. 14-4875-82/Dan11mag), rabbit polyclonal anti-POU3F1 (1:100, Abcam, catalog no. ab272925), and rabbit monoclonal anti-CTIP2 (1:250, Abcam, catalog no. ab240636). The following secondary antibodies were used: goat polyclonal anti-mouse 488/546/647 (1:500, Thermo Fisher Scientific, catalog no. A-11006/A-11030/A-21236) and goat polyclonal anti-rabbit 488/546/647 (1:500, Thermo Fisher Scientific, catalog no. A-11034/A-11035/A-21245).

### Image acquisition

Confocal image acquisition was performed using inverted confocal microscopes LSM 800 series (Zeiss), SP8, or Stellaris5 (Leica). Optical z-sections were obtained by taking 1- to 3.5-μm serial confocal images with LAS AF v1.8 software (Leica) or ZEN Blue 3.8 (Zeiss). Mosaic images were obtained with the 20×/0.8 objective at 512 by 512 or 1024 by 1024 resolution (8 bits). High-magnification images were acquired with the 63×/1.4 oil immersion objective at 1024 by 1024 resolution (8 bits). All images were analyzed using Fiji-ImageJ (*77*) and ZEN Blue 3.8 (Zeiss). For MADM clones, sections were first screened using an Axioscope (Zeiss Axio Imager, Zeiss) coupled to a CoolLED p300 SB light source (CoolLED) and equipped with 10×/0.45 and 20×/0.8 objectives (Zeiss). Green and red fluorescence were observed using an HC-dualband GFP/DsRed filter (F56-420, AHF).

### Quantification of IUE and EdU, CTB-labeled populations

To delimit equivalent S1-barrel field (Bf) areas in the developing brain across stages, we used the Developing Mouse Reference Atlas from Allen Brain Atlas (http://atlas.brain-map.org/) and anatomical landmarks. For EdU, IUE, and injections in the IC, CerbPed, or dorsal striatum experiments, the entire S1-Bf region was quantified after the area and cortical layers were outlined based on anatomical landmarks and their distinct cell densities. To quantify CTB^+^ cells, we analyzed confocal plane z-stack images spanning the width of each cell of interest (*33*, *35*). Cells are marked as positive if the CTB fluorescent signal surrounds the DAPI nuclei of the analyzed cell (NeuN^+^, EdU^+^, and GFP^+^). For CTB^+^NeuN^+^ quantification after injections in the CC, 250 NeuN^+^ cells (50 per layer) were randomly selected within a region of interest (ROI) depicting a column in the S1-Bf on the hemisphere contralateral to the injection site. Data are provided as the percentage of CTB^+^ neurons out of NeuN^+^ cells. In animals injected with two different CTBs in the CC and IC or CerbPed, and with EdU^+^, the percentages were calculated from the sum of all EdU+ cells labeled with any of the CTBs in S1-Bf area. For the estimates of the fractions of PNs born at the different embryonic stages, fractions were calculated from the total number of EdU^+^ CTB^+^ in each layer or in the totality of S1-Bf across all stages. In electroporated animals, percentages were calculated from the total number of GFP^+^ cells in the S1-Bf area. In embryonic brain sections, a division index (EdU dilution rate) was calculated from the proportion of EdU^+^ and EdU^−^ cells among GFP^+^ (electroporated) cells quantified within a 250-μm ROI. A minimum of two sections (technical replicates) were analyzed per individual brain. At least three animals (biological replicates) were analyzed per condition/stage.

### Analysis of the single-cell RNA seq dataset

To cluster RGPs and study differential gene expression over developmental time, we analyzed the single-cell RNA sequencing atlas of the developing neocortex published by Di Bella *et al.* (*34*) (datasets in the Gene Expression Omnibus SuperSeries GSE153164). We selected cortical cells from E12.5 to E15.5 samples. The analysis included the following steps using R software (DOI: 10.5281/zenodo.14609057): (i) Normalization and filtering: Counts were standardized for each time point through the normalization of the feature expression measurements for each cell by the total expression, scaling by a factor of 10,000, and applying a log+1 transformation. The dataset was filtered to include only samples from embryonic stages E12 to E15. Before this analysis, the data were already prefiltered to retain high-quality cells, with mitochondrial reads <7.5% and detected genes >500. (ii) Feature selection and scaling: We identified the 3000 most variable features using the FindVariableFeatures function with the “vst” selection method. During scaling, we regressed out variability associated with “nCount_RNA” and “nFeature_RNA” to correct for technical artifacts related to RNA capture and sequencing depth. (iii) Dimensionality reduction and clustering: We performed linear dimensionality reduction using principal components analysis (PCA) on the scaled data, retaining 50 principal components. A *k*-nearest-neighbors graph was constructed based on Euclidean distance in PCA space using these 50 components (FindNeighbors, dims = 1:50). Cells were clustered using the Louvain algorithm implemented in Seurat, with a resolution parameter set to 0.7 (FindClusters). (iv) Visualization: For visualization, Uniform Manifold Approximation and Projection (UMAP) was applied to the first 15 principal components (RunUMAP). (v) Cell annotations and differential expression: Cell annotations for embryonic stage and cell type were incorporated into the analysis. Differentially expressed genes were identified using FindAllMarkers in Seurat with the Wilcoxon Rank Sum test, applying a Bonferroni correction for multiple testing (adjusted *P* value <0.05). Only genes detected in at least 25% of the cells within a cluster and showing a minimum 0.25-fold difference (log-scale) between the cells in the cluster and all remaining cells were considered.

### Statistical analysis

All experimental conditions include a minimum of three mice and two coronal sections per brain, except for MADM experiments in which we analyzed the entire somatosensory cortex in each brain. Only the animals in which CTB labeling was not efficient or in which IUE experiments targeted other cortical areas than the somatosensory cortex were excluded from the analysis. Results show the sample mean ± SEM or the median. The statistical groups were determined by embryonic and/or postnatal experimental day, treatment, or layer. Sample normality was verified using the D’Agostino-Pearson or Shapiro-Wilk test. Data visualization was performed using GraphPad Prism 10.0 (GraphPad Software). The results were compared using different tests depending on the study object and the number of variables. The Student’s *t* test was used when comparing the mean values of one variable between two groups; one- or two-way analysis of variance (ANOVA), followed by a post hoc test, Dunnett’s, Tukey’s, or Šídák’s multiple comparisons test as specified in the figure legends. Kruskal-Wallis test or Wilcoxon matched-pairs tests were used for samples that did not follow a normal distribution. Fisher exact test and chi-square test were applied to compare distributions. Linear regression and correlation (Spearman test) analysis were used to evaluate the relationship between variables. Statistically significant differences were established at *P < 0.05, **P < 0.01, ***P < 0.001, and ****P < 0.0001, “n.s.” stands for nonsignificant.

Chi-square and permutation tests were combined to assess the significance of the association between clone/subclone type (IT-PN only, ET-PN only, and Mixed) and the type of division of the clone/subclone (Proliferative, Asymmetric, and Terminal). A chi-square statistic was calculated for the observed data and then compared to chance using the R function “chisq.test.” The “type of clone/subclone” column was permuted 1000 times, and a chi-square statistic was calculated for each permutated dataset. The *P* value was calculated as the proportion of permuted chi-square statistics that are greater than or equal to the observed chi-square statistic.

Estimates of death rate in MADM clones and of the maximal potential frequency of IT-PN clones emerging from the death of ET-PNs in a Mixed clone: The cell death rate (probability = 0.08 per cell) was calculated based on the frequency of orphan/single-color subclones, as previously described (*8*). This value aligns with earlier reports (*8*, *15*). For the estimate of misclassification errors due to cell death, we considered that there could be “ghost”-Mixed clone/lineage misclassified as pure clone/lineage due to the cell death of ET-PNs or IT-PNs. The maximum frequency of a ghost-mixed clone/lineage was calculated considering that, in a Mixed lineage, the frequency of losing ET-PN or IT-PN branches cannot exceed the progenitor death rate. Thus, we calculated a maximum error of our measurements as the probability of the death of either the ET-PN or IT-PN progenitor in a Mixed clone or lineage. For example, the frequency of Mixed clones in the population labeled at E12.5 is 0.78. Thus, the maximum frequency of death of ET-PN or IT-PN precursor in a Mixed clone/lineage = (frequency of Mixed clones/lineage) × 0.08 (death rate of a subclone) × 0.5 (probability of ET-PN or IT-PN). The errors we obtained exclude statistically the possibility that the frequencies of pure clones and lineages we observed are due to cell death. Examples are as follows:

• Error in the measurement of E12.5 ET-PN clones:

Maximum frequency of death of ET-PN precursor in a Mixed clone/lineage = (frequency of Mixed clones/lineage) × 0.08 (death rate of a subclone) × 0.5 (probability of ET-PN) = 0.03 (3%), which provides a final frequency of 10.6 ± 3%.

• Error in the measurements of E12.5 ET-PN lineages:

Maximum frequency of death of ET-PN precursor in lineage = 0.48 (frequency of Mixed lineages) × 0.08 × 0.5 = 0.02 (2%), which provides a final frequency of 24 ± 2%.

• Error in the measurements of E12.5 IT-PN lineages:

Maximum frequency of death of IT-PN precursor in a Mixed clone/lineage = (frequency of Mixed clones/lineage) × 0.08 (death rate of a subclone) × 0.5 (probability of IT-PN) = 0.03 (3%). The experimental frequency of E12.5 IT-PN clones is 12%, which, with the error accounting for cell death, provides a final frequency of 12 ± 3%.

Alternative statistical calculations including additional considerations provide even lower error rates due to cell death. For instance, by considering the size of the clone/lineage, we can refine our estimates: e.g., in an IT-PN clone of six neurons, the error due to the death of an ET-PN in a Mixed clone of seven neurons can be calculated using the experimental frequency of an IT-PN clone with a size of seven and the probability of the dying cell being an ET-PN (1/8). This yields an error frequency for clones/lineages of eight neurons of 0.000196 (0.0196%).

## Supplementary Materials

### The PDF file includes:

Figs. S1 to S9 Legend for data S1 Annex S1-MADM Atlas
