# Supplementary material for: Early fate diversification of radial glial progenitors during corticogenesis
Source: Sci Adv. 2026 Aug 5;12(32):eadw5487. doi: 10.1126/sciadv.adw5487 (PMC13440428; doi:10.1126/sciadv.adw5487)

Science Advances
AAAS

Supplementary Materials for

Early fate diversification of radial glial progenitors during corticogenesis

Irene Varela-Martínez *et al.*

Corresponding author: Marta Nieto, mnlopez@cnb.csic.es

Sci. Adv. **12**, eadw5487 (2026)
DOI: 10.1126/sciadv.adw5487

The PDF file includes:

Figs. S1 to S9
Legend for data S1
Annex S1-MADM Atlas

Other Supplementary Material for this manuscript includes the following:

Data S1

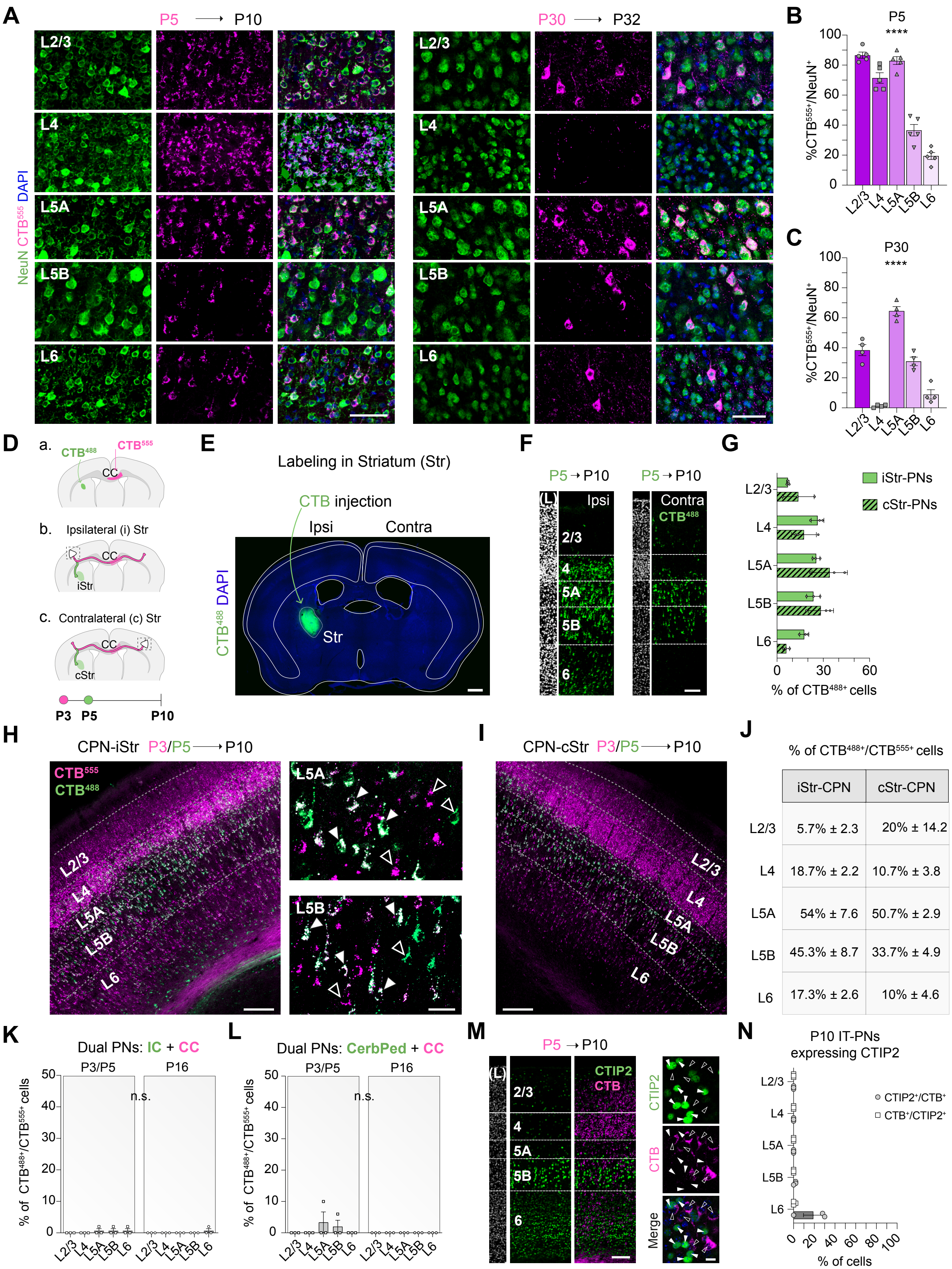

	iStr-CPN	cStr-CPN
L2/3	5.7% ± 2.3	20% ± 14.2
L4	18.7% ± 2.2	10.7% ± 3.8
L5A	54% ± 7.6	50.7% ± 2.9
L5B	45.3% ± 8.7	33.7% ± 4.9
L6	17.3% ± 2.6	10% ± 4.6

Fig. S1.

Developmental callosal projections identify IT-PNs, related to Fig. 1 and 2

(**A**) NeuN (green), CTB-555 (magenta), and DAPI (blue) signals in the ULs and DLs of brains injected and analyzed at the indicated times. The stage shown in magenta indicates the time of injection; the stage shown in black indicates the time of analysis. (**B** and **C**) Percentages of CPNs (CTB-555$^+$NeuN$^+$) quantified over 50 NeuN+ per layer. CTB injections in the CC were performed at P5 (**B**) or P30 (**C**). Mean ± SEM (n = 500 cells, n = 2 sections/animal; n ≥ 4 animals). One-way ANOVA (CTB$^+$NeuN$^+$ across layers): **** p-value <0.0001. (**D**) Schematic illustrating the analysis of PNs projecting dually to both the CC and to the striatum (Str): (a) CTB-555 (magenta) was injected into the CC at P3 followed by CTB-488 (green) injections into the Str at P5; (b) representation of a PN projecting ipsilaterally to the Str (iStr) and callosally; (c) representation of a CPN projecting to the contralateral Str (cStr). (**E**) CTB-488 signal confined within the dorsal-Str two hours post-injection. (**F**) CTB-488 labeling of S1-Bf PNs projecting to the ipsilateral (iStr-PN, left) or contralateral (cStr-PN, right) Str following injections as outlined in (**E**). (**G**) Percentage of CTB-488+ iStr-PNs and cStr-PNs following injections into the Str as in (**E**). Mean ± SEM (n = 2 sections/animal, n = 3 animals). (**H** and **I**) S1-Bf cortices ipsilateral (**H**) or contralateral (**I**) to the striatal injections after injections as described in (**D**). High-magnifications in (**H**) show L5A and L5B dually labeled cells (white arrowheads: callosal and cortico-striatal) and single-labeled cells (empty arrowheads), exclusively cortico-striatal or exclusively callosal. (**J**) Percentages of CTB-488+ (striatal injection) over CTB-555$^+$ (callosal injection) cells per layer. Mean ± SEM (n = 500 cells/hemisphere, n = 2 sections/animal, n = 3 animals). (**K** and **L**) Percentage of CTB-488$^+$ over CTB-555$^+$ cells after IC (**K**) or CerbPed (**L**) injections as in main Fig. 2B. Mean ± SEM (n = 500 cells, n = 2 sections/animal; n = 3 animals/group). The two-way ANOVA analysis, comparing with a theoretical zero, showed non-significant main effects or interactions. (**M**) Immuno-labeling of CTIP2 (green) in P10 coronal brain slices after animals were injected with CTB in the CC at P5 (magenta). Low-magnification (left) and high-magnification images (right). White arrowheads indicate CTIP2$^+$ cells, empty arrowheads indicate CTB+ PNs, showing no co-labeling. (**N**) Percentage of CTIP2$^+$ over CTB$^+$ PNs (gray circles) and CTB+ over CTIP2$^+$ PNs (white squares) per layer. Mean ± SEM (n = 250 cells per quantification, n = 3 animals). **Scale Bars:** 50 µm (A, F), 500 µm (E), 300 µm (H, I, low magnification), 150 µm (H, high magnification), 100 µm (M, low magnification), 10µm (M, high magnification).

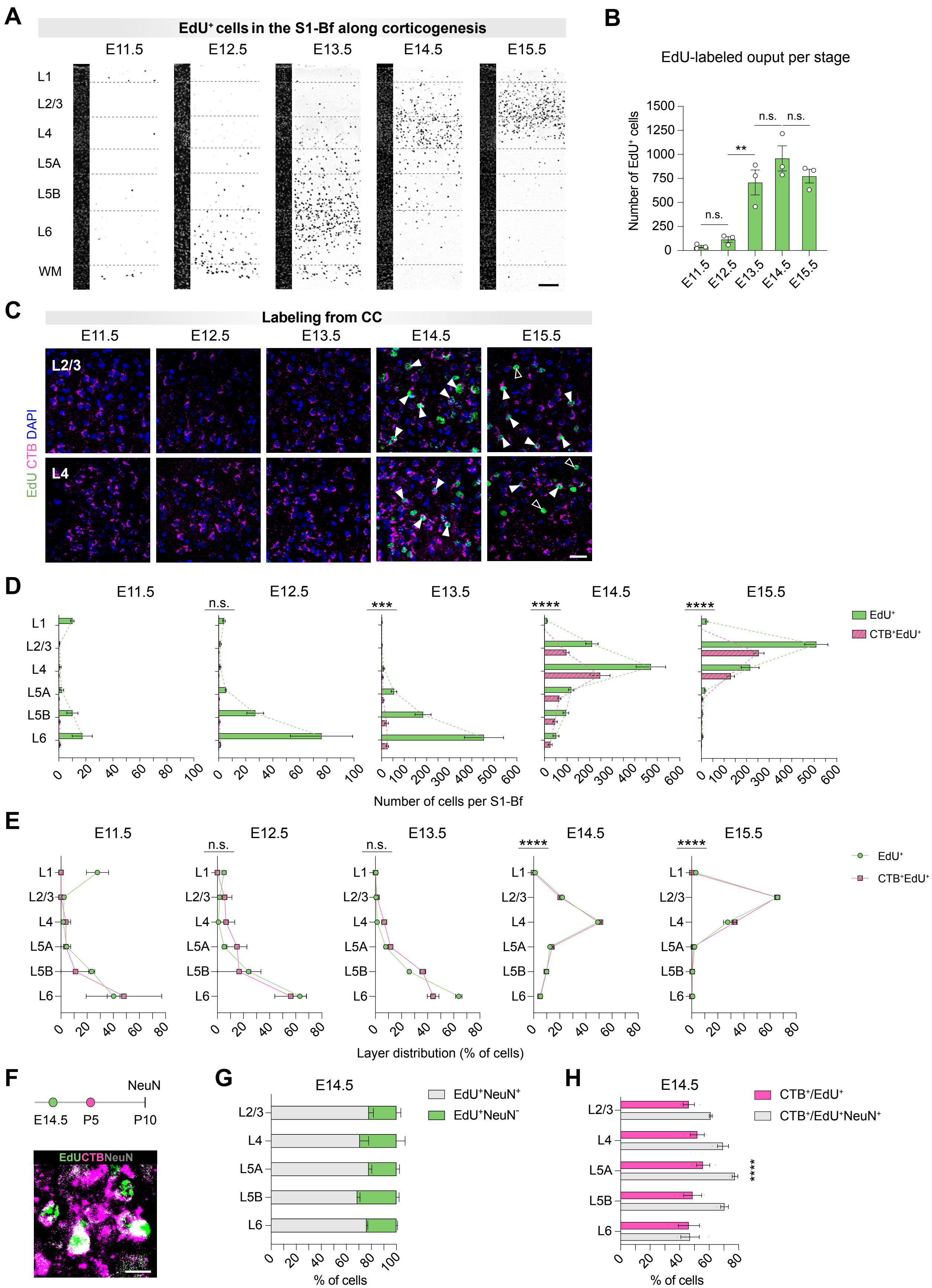
A
EdU+ cells in the S1-Bf along corticogenesis
E11.5
E12.5
E13.5
E14.5
E15.5
L1
L2/3
L4
L5A
L5B
L6
WM
B
EdU-labeled ouput per stage
Number of EdU+ cells
n.s.
**
n.s.
n.s.
C
Labeling from CC
EdU CTB DAPI
L2/3
L4
D
E11.5
E12.5
E13.5
E14.5
E15.5
n.s.
***
****
****
EdU+
CTB+EdU+
Number of cells per S1-Bf
E
Layer distribution (% of cells)
F
NeuN
E14.5
P5
P10
EdU CTB NeuN
G
E14.5
EdU+NeuN+
EdU+NeuN-
% of cells
H
E14.5
CTB+/EdU+
CTB+/EdU+NeuN+
****
% of cells

Fig. S2.

Birth-date analysis of IT-PNs using EdU injections, related to Fig. 3

(**A**) EdU labeling (black) in the S1-Bf at P10 following EdU injections at the indicated embryonic stages. Dashed lines outline cortical layers. (**B**) Total numbers of EdU^+ cells in the S1-Bf at P10 following intraperitoneal EdU injections at the indicated stages. Mean ± SEM (n = 2 sections/animal, n = 3 animals/stage). One-way ANOVA test with Tukey's post-hoc test (comparisons were made between consecutive stages): ** p-value $_{E12.5\ vs\ E13.5}$ =0.006. (**C**) CTB-555 (magenta) and DAPI (blue) signals at P10 in the S1-Bf ULs following EdU injections at the indicated embryonic stages and CTB injection in the CC at P5. Absence of EdU signal (green) indicates no UL-PN generation during E11.5, E12.5 and E13.5 stages. White arrowheads indicate double CTB^+EdU^+ cells, empty arrowheads indicate CTB-EdU^+ PNs. (**D**) Absolute numbers of birth-dated EdU^+ cells (green) and IT-PNs (dashed magenta) in the S1-Bf at P10 following EdU injections at the indicated embryonic stages and CTB injection in the CC at P5. Mean ± SEM (n= 2 sections/animal; n = 3 animals/stage). Dashed line connects the mean values of each layer. Two-way ANOVA (CTB^+EdU^+ across consecutive stages, Stage x Layer): *** p-value =0.0005 $_{E12.5-E13.5}$, **** p-value <0.0001 $_{E13.5-E14.5}$, **** p-value<0.0001 $_{E14.5-E15.5}$. (**E**) Preferred layer of migration of birth-dated cells. Data derived from (**D**). Values represent percentage of CTB^+EdU^+ cells per layer in S1-Bf. Mean ± SEM (n = 2 sections/animal; n = 3 animals/stage). Two-way ANOVA (CTB^+EdU^+ across consecutive stages, Stage x Layer): **** p-value <0.0001 $_{E13.5-E14.5}$, **** p-value <0.0001 $_{E14.5-E15.5}$. (**F**) Experimental design (top) and high-magnification image (bottom) showing EdU (green), CTB (magenta) and NeuN (white) colabeling (**G**) Proportion of neuronal ($NeuN^+$) and non-neuronal ($NeuN^-$) newborn cells per layer labeled with EdU at E14.5. Percentages were calculated from the total number of cells in S1-Bf. Mean ± SEM (n = 2 sections/animal; n = 3 animals). (**H**) Percentage of IT-PNs (CTB^+) at P10 calculated over EdU^+ labeling (bars in magenta) or EdU^+NeuN^+ labeling (bars in gray). EdU injections were performed at E14.5 and CTB injections into the CC at P5. Mean ± SEM (n = 2 sections/animal; n = 3 animals). Two-way ANOVA ($_{CTB^+EdU^+\ vs.\ CTB^+EdU^+NeuN^+}$): **** p < 0.0001), Condition x Layer non-significant. **Scale bars:** 100 μm (A), 20 μm (C, F).

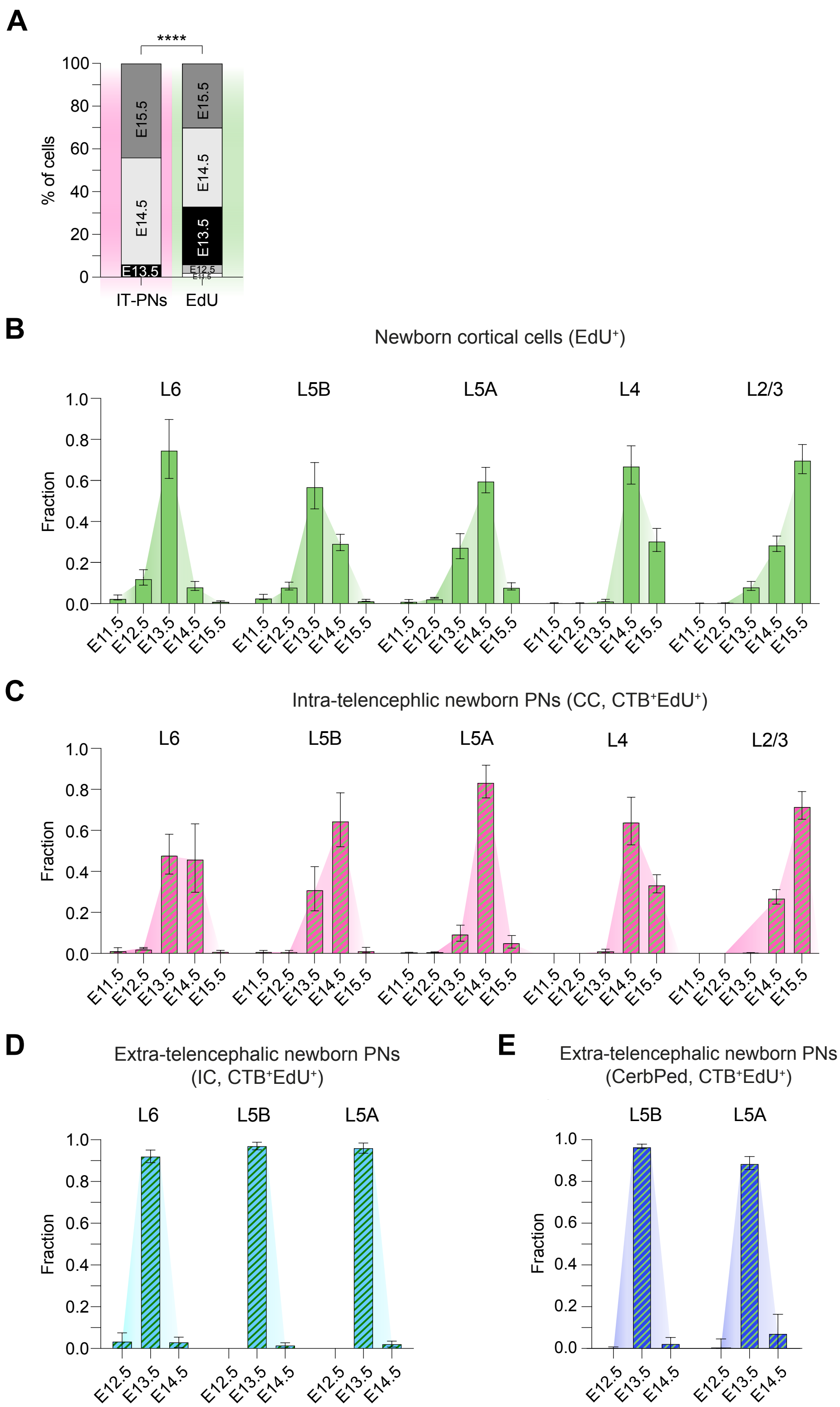

A
****
% of cells
E15.5
E14.5
E13.5
E12.5
IT-PNs
EdU
B
Newborn cortical cells (EdU⁺)
L6
L5B
L5A
L4
L2/3
Fraction
E11.5
E12.5
E13.5
E14.5
E15.5
C
Intra-telencephlic newborn PNs (CC, CTB⁺EdU⁺)
D
Extra-telencephalic newborn PNs
(IC, CTB⁺EdU⁺)
E
Extra-telencephalic newborn PNs
(CerbPed, CTB⁺EdU⁺)

Fig. S3.

Distinct neurogenic timing of cortical PN subtypes, related to Fig. 3

(**A**) Distribution of CTB^+ EdU^+ (IT-PNs) and EdU^+ cells born across the indicated stages. Data were calculated using the averages of the total number of IT-PNs or EdU^+ cells per embryonic stage: (n = 2 sections/animal, n = 3 animals/stage). Chi-square test: **** p-value $_{\text{IT-PNs vs. EdU}}$ <0.0001. (**B-E**) Fraction of EdU^+ cells (**B**) and CTB^+ EdU^+ PNs (**C**, IT-PNs; **D**, IC ET-PNs; **E**, CerbPed ET-PNs) born in the S1-Bf at the indicated stages per layer. Percentages were calculated from the total number of cells in S1-Bf. Mean ± SEM (n= 2 sections/animal, n ≥ 3 animals/stage).

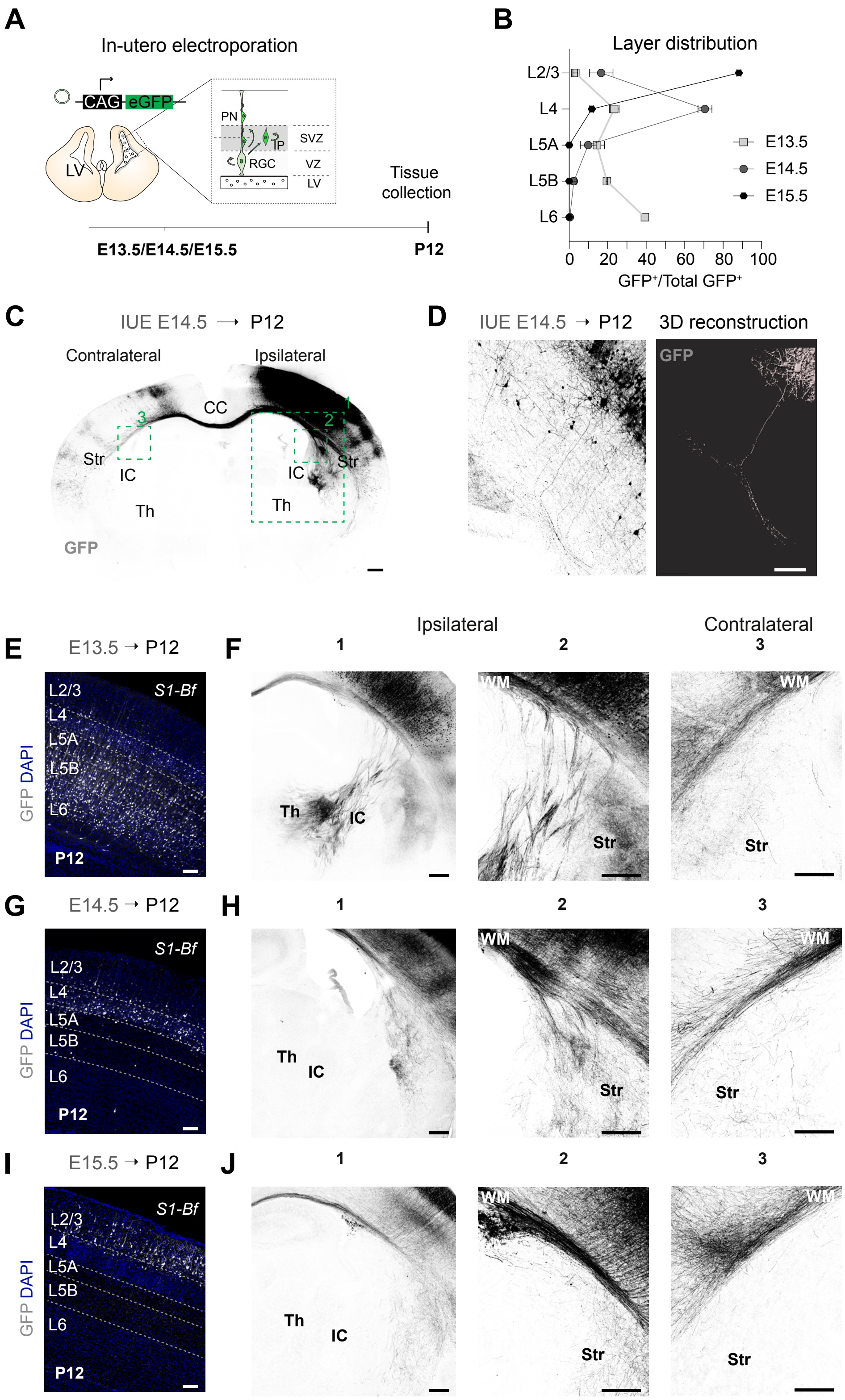

A
In-utero electroporation
CAG eGFP
PN
IP
SVZ
RGC
VZ
LV
LV
Tissue collection
E13.5/E14.5/E15.5
P12
B
Layer distribution
L2/3
L4
L5A
L5B
L6
E13.5
E14.5
E15.5
0 20 40 60 80 100
GFP⁺/Total GFP⁺
C
IUE E14.5 → P12
Contralateral
Ipsilateral
CC
Str
IC
Th
GFP
D
IUE E14.5 → P12
3D reconstruction
GFP
E
E13.5 → P12
S1-Bf
GFP DAPI
P12
F
Ipsilateral
Contralateral
WM
Th
IC
Str
G
E14.5 → P12
H
I
E15.5 → P12
J

Fig. S4.

IUE confirmed that ET-PNs are not generated during late neurogenesis, related to Fig. 3 (**A**) Schematic of the experimental design. Dividing progenitors in the VZ were electroporated at E13.5, E14.5, or E15.5 with a GFP-encoding plasmid, followed by analysis at P12. (**B**) Layer distribution of GFP^+ cells in the S1-Bf at P12. Percentages were calculated from the total number of GFP^+ cells in S1-Bf. Mean ± SEM (n = 2 sections/ animal; n = 3 animals/embryonic stage). (**C**) GFP signal following IUE at E14.5. Green boxes indicate regions of interest: (1) ipsilateral IC and Th, (2) ipsilateral dorsal-Str and WM, (3) contralateral dorsal-Str and WM. Corresponding high magnification images are shown in panels (**E**) for E13.5, (**F**) for E14.5, and (**I**) for E15.5. (**D**) High-magnification image (left) and 3D Imaris reconstruction of an L5A PN extending a dual projection to the CC and the ipsilateral Str (right). (**E, G**, and **I**) S1-Bf of P12 brains showing GFP^+ electroporated neurons following IUE at E13.5 (**E**), E14.5 (**F**), or E15.5 (**G**). Cortical layers are outlined by dashed lines. (**F, H,** and **J**) High-magnification images showing GFP^+ axons within the regions indicated in (**C**) following IUE at E13.5 (**F**), E14.5 (**H**), and E15.5 (**J**). **Scale bars:** 1 mm (C), 150 µm (D), 100 µm (E, G, I), 250 µm (F, H, J). Abbreviations: CC: Corpus Callosum, IC: Internal Capsule, IP: Intermediate Progenitor, LV: Lateral Ventricle, PN: Projection neuron, RGP: Radial Glial Cell, Str: Striatum, SVZ: Subventricular Zone, Th: Thalamus, VZ: Ventricular Zone, WM: White Matter.

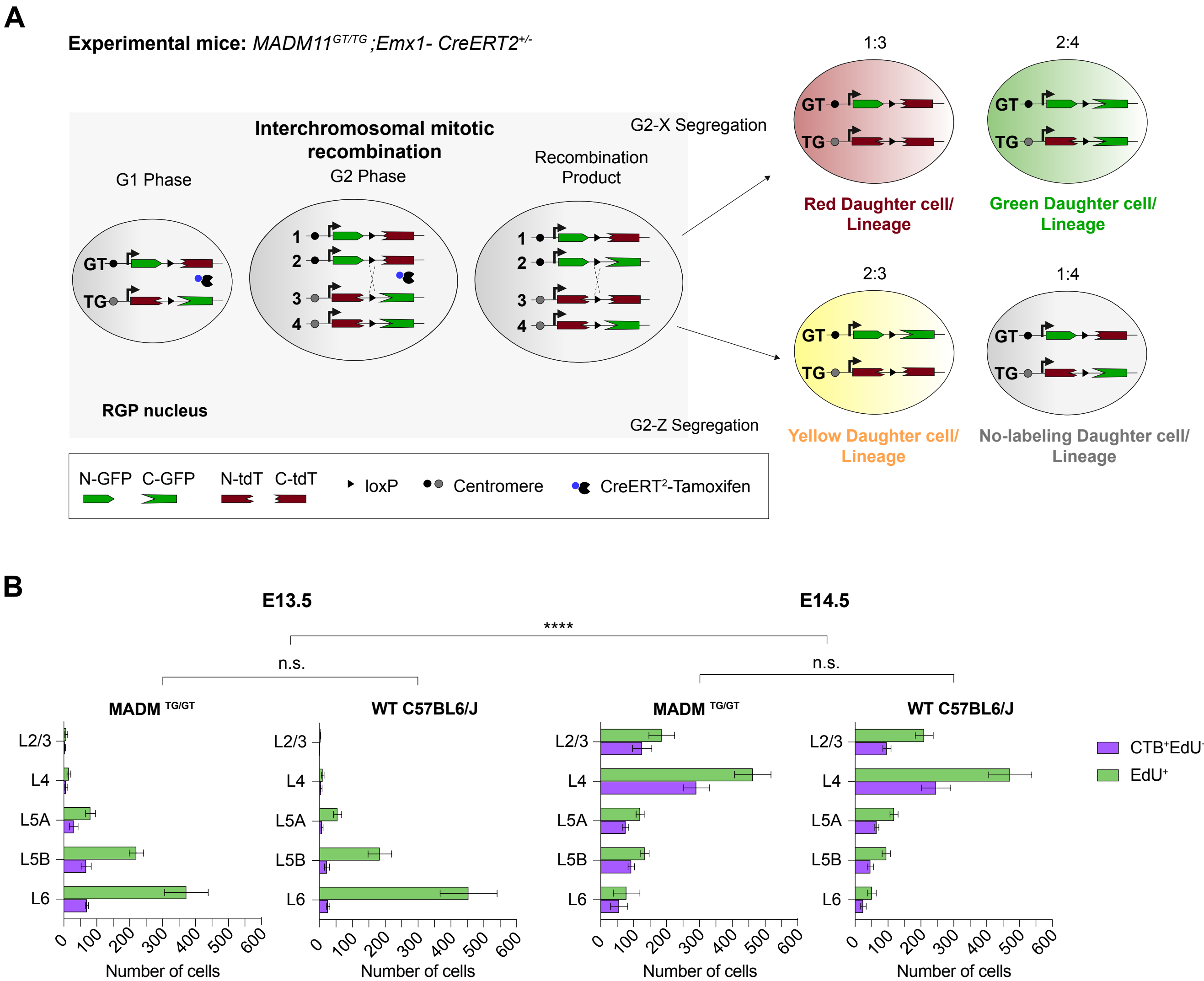

A
Experimental mice: MADM11GT/TG ;Emx1- CreERT2+/-
Interchromosomal mitotic recombination
G1 Phase
G2 Phase
Recombination Product
G2-X Segregation
G2-Z Segregation
RGP nucleus
1:3
2:4
2:3
1:4
Red Daughter cell/ Lineage
Green Daughter cell/ Lineage
Yellow Daughter cell/ Lineage
No-labeling Daughter cell/ Lineage
N-GFP C-GFP N-tdT C-tdT loxP Centromere CreERT2-Tamoxifen
B
E13.5
E14.5
****
n.s.
n.s.
MADM TG/GT
WT C57BL6/J
L2/3
L4
L5A
L5B
L6
Number of cells
CTB+EdU+
EdU+

Fig. S5.

MADM principle and EdU birth-dating analysis in MADM mice, related to Fig. 4
(**A**) Schematic of MADM labeling. Males and females of Emx1-CreERT2$^{+/-}$ and *MADM11*$^{GT/TG}$ genotypes were used indistinctly. (**B**) Absolute numbers of EdU^{+} cells (green) and CTB^{+}EdU^{+} cells (magenta; IT-PNs) in the S1-Bf at P10 following EdU injections at the indicated embryonic stages and CTB injection in the CC at P5. Mean ± SEM ($n \geq 3$ animals/stage). Two-way ANOVA ($_{\text{CTB+EdU+ MADM vs. CTB+EdU+ WT}}$) showed no significant main effects. Three-way ANOVA ($_{\text{E13.5MADM-WT vs E14.5MADMWT}}$): **** p-value <0.0001.

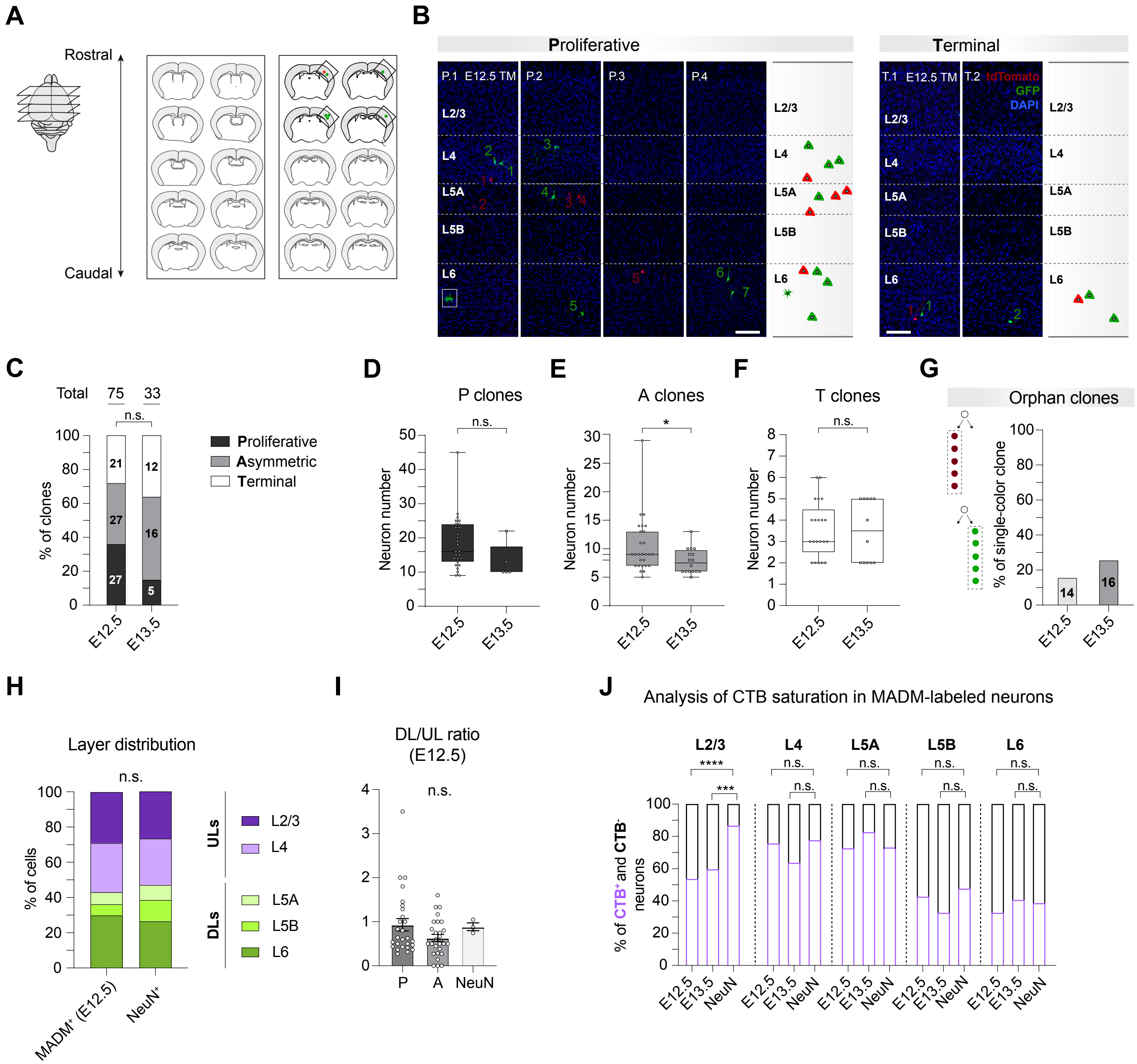
A
Rostral
Caudal
B
Proliferative
Terminal
P.1 E12.5 TM
P.2
P.3
P.4
T.1 E12.5 TM
T.2
tdTomato
GFP
DAPI
L2/3
L4
L5A
L5B
L6
C
Total 75 33
n.s.
% of clones
Proliferative
Asymmetric
Terminal
E12.5
E13.5
D
P clones
E
A clones
F
T clones
Neuron number
G
Orphan clones
% of single-color clone
H
Layer distribution
% of cells
ULs
DLs
L2/3
L4
L5A
L5B
L6
MADM+ (E12.5)
NeuN+
I
DL/UL ratio
(E12.5)
P
A
NeuN
J
Analysis of CTB saturation in MADM-labeled neurons
% of CTB+ and CTB- neurons

Fig S6.

MADM-based clonal analysis in combination with CTB axonal tracing, related to Fig. 4

(**A**) Schematic of sequential brain section preparation for MADM clone analysis. (**B**) Consecutive brain sections of Proliferative and Terminal G2-X clones. Each clone contains a red (tdTomato) and a green (GFP) subclone. PN numbers/subclone in corresponding colors. Right-most panels: schematics of the reconstructed clones. Scale bars: 100 µm. (**C**) Proportion of clones in each category following TM induction at E12.5 or E13.5. Chi-square test: p-value=0.0915. Bold numbers represent the number of clones per category. (**D-F**) Clone sizes for Proliferative (**D**), Asymmetric (**E**), and Terminal (**F**) clones (box, median with 25-75 percentiles; whiskers, minimum and maximum values). Dots represent neurons per individual clone (n = number of clones per category and stage indicated in **C**). Mann-Whitney test (E12.5 vs. E13.5): (**D**) p-value =0.0655, (**E**) * p-value =0.037, (**F**) p-value=0.8672. (**G**) Percentage of single color/orphan clones. The probability of death is inferred as the proportion of single-color (tdTomato$^+$ or GFP$^+$) clones in the population (15.5%). (**H**) Fraction of MADM$^+$ cells (following TM induction at E12.5) or NeuN$^+$ cells (within a 500 µm ROI in P10 brains) across cortical layers. Data represent the average distribution of all MADM+ E12.5 cells versus the average distribution of NeuN$^+$ cells quantified within the same ROI in three independent animals. Chi-square test for comparison showed non-significant differences (n.s.; p-value $_{\text{MADM vs NeuN}}$ =0.6680). (**I**) DL to UL ratio in proliferative (**P**) and asymmetric (**A**) clones, or NeuN+ population. Mean ± SEM (n = 27 proliferative clones, n = 27 asymmetric clone; n = NeuN+ cells in a 500 µm ROI, n = 3 animals). One-way ANOVA analysis showed non-significant main effects. (**J**) Proportion of CTB$^+$ (magenta) and CTB- (black) neurons per layer in MADM$^+$ and NeuN$^+$ populations at P10 following CTB injection in the CC at P5. Chi-square test: (L2/3) **** p-value $_{\text{E12.5 MADM+ vs P5 NeuN+}}$ <0.0001, *** p-value E13.5 $_{\text{MADM+ vs P5 NeuN+}}$ =0.0003. Proportions of CTB$^+$NeuN$^+$ were calculated using the data in Fig. 1.

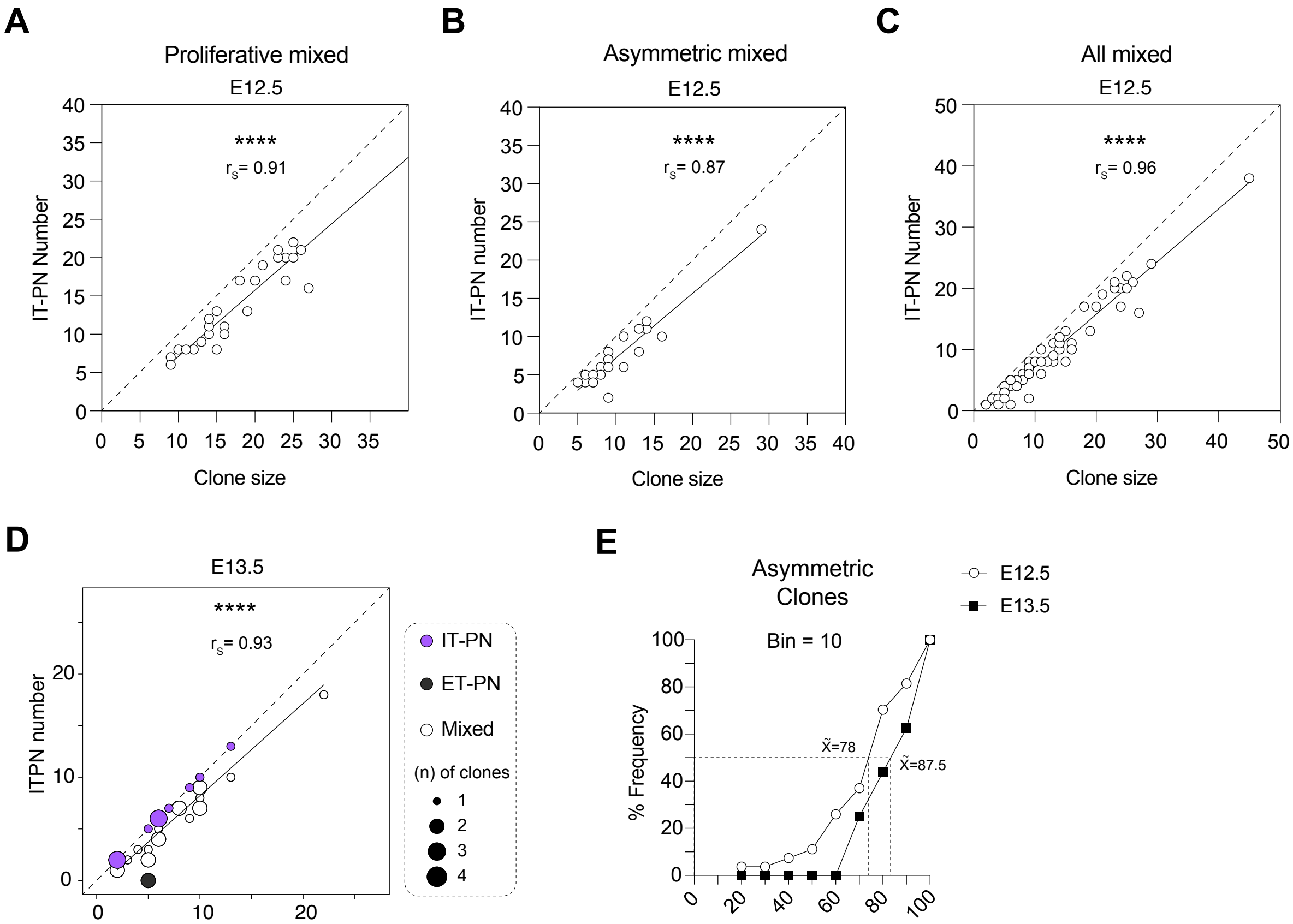
A
Proliferative mixed
E12.5
****
r_s= 0.91
IT-PN Number
Clone size
B
Asymmetric mixed
E12.5
****
r_s= 0.87
IT-PN number
Clone size
C
All mixed
E12.5
****
r_s= 0.96
IT-PN Number
Clone size
D
E13.5
****
r_s= 0.93
ITPN number
IT-PN
ET-PN
Mixed
(n) of clones
1
2
3
4
E
Asymmetric Clones
Bin = 10
% Frequency
X̃=78
X̃=87.5
E12.5
E13.5

Fig. S7.

Scaling relationship between IT-PN number and clone size, related to Fig. 5

(**A-C**) Plot comparing IT-PN number vs clone size in proliferative mixed (**A**), asymmetric mixed (**B**), and all mixed clones (including terminal clones) (**C**) for clones labeled at E12.5. The black line represents linear regression: (**A**) y = 0.8666x Clone size– 1, (**B**) y = 0.8450x Clone size – 1.250, (**C**) y = 0.8615x Clone size – 1, where y is IT-PN number. Spearman correlation (rs=Spearman's rank correlation coefficient): (**A**) rs (26) = 0.9105, (**B**) rs (22) = 0.8650, (**C**) rs (58) = 0.9611; (**A-C**) **** p-value<0.0001. (**D**) Plot of IT-PN number vs. clone size for clones labeled at E13.5. The bisector (dashed line) indicates IT-PN clones. The black continuous line represents linear regression: y = 0.8955x Clone size – 0.7, where y is IT-PN number. Spearman correlation (rs =Spearman's rank correlation coefficient): rs (33) = 0.93, **** p-value <0.0001. (**E**) Cumulative frequency of asymmetric clones labeled at E12.5 (white circle) or E13.5 (black square) with increasing IT-PN Bin-percentages (Bin=10). Intersection with dashed lines represents the median (E12.5 n = 27 clones, E13.5 n = 16 clones).

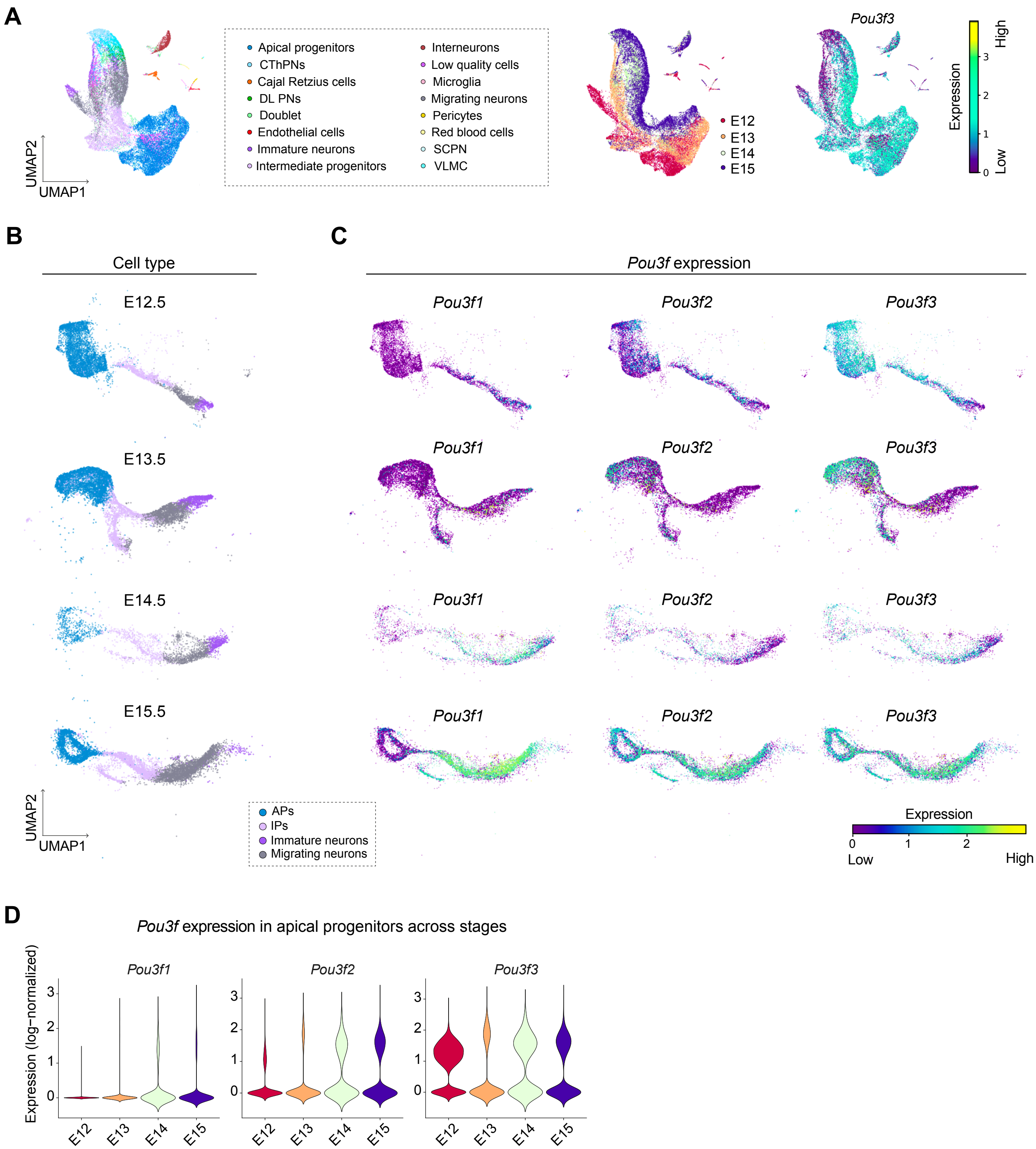
A
UMAP2
UMAP1
Apical progenitors
CThPNs
Cajal Retzius cells
DL PNs
Doublet
Endothelial cells
Immature neurons
Intermediate progenitors
Interneurons
Low quality cells
Microglia
Migrating neurons
Pericytes
Red blood cells
SCPN
VLMC
E12
E13
E14
E15
Pou3f3
Expression
High
Low
B
Cell type
E12.5
E13.5
E14.5
E15.5
APs
IPs
Immature neurons
Migrating neurons
C
Pou3f expression
Pou3f1
Pou3f2
Pou3f3
Expression
Low
High
D
Pou3f expression in apical progenitors across stages
Expression (log-normalized)

Fig. S8.

Gene expression analysis of POU factors during neurogenesis, related to Fig. 6
(**A**) UMAP plots showing annotated cell populations across developmental stages (E12.5-E15.5) (left). Expression pattern of Pou3f3 in the same dataset (right). (**B** and **C**) UMAP plot colored by (**B**) cell type or (**C**) Pou3f gene expression across cell types, in the indicated stages. (**D**) Violin plots showing the expression of Pou3f1, Pou3f2 and Pou3f3 in apical progenitors (blue in panel **B**) across developmental time points. (**A**-**D**) Single-cell RNA sequencing data from *Di Bella et al.,* (20).

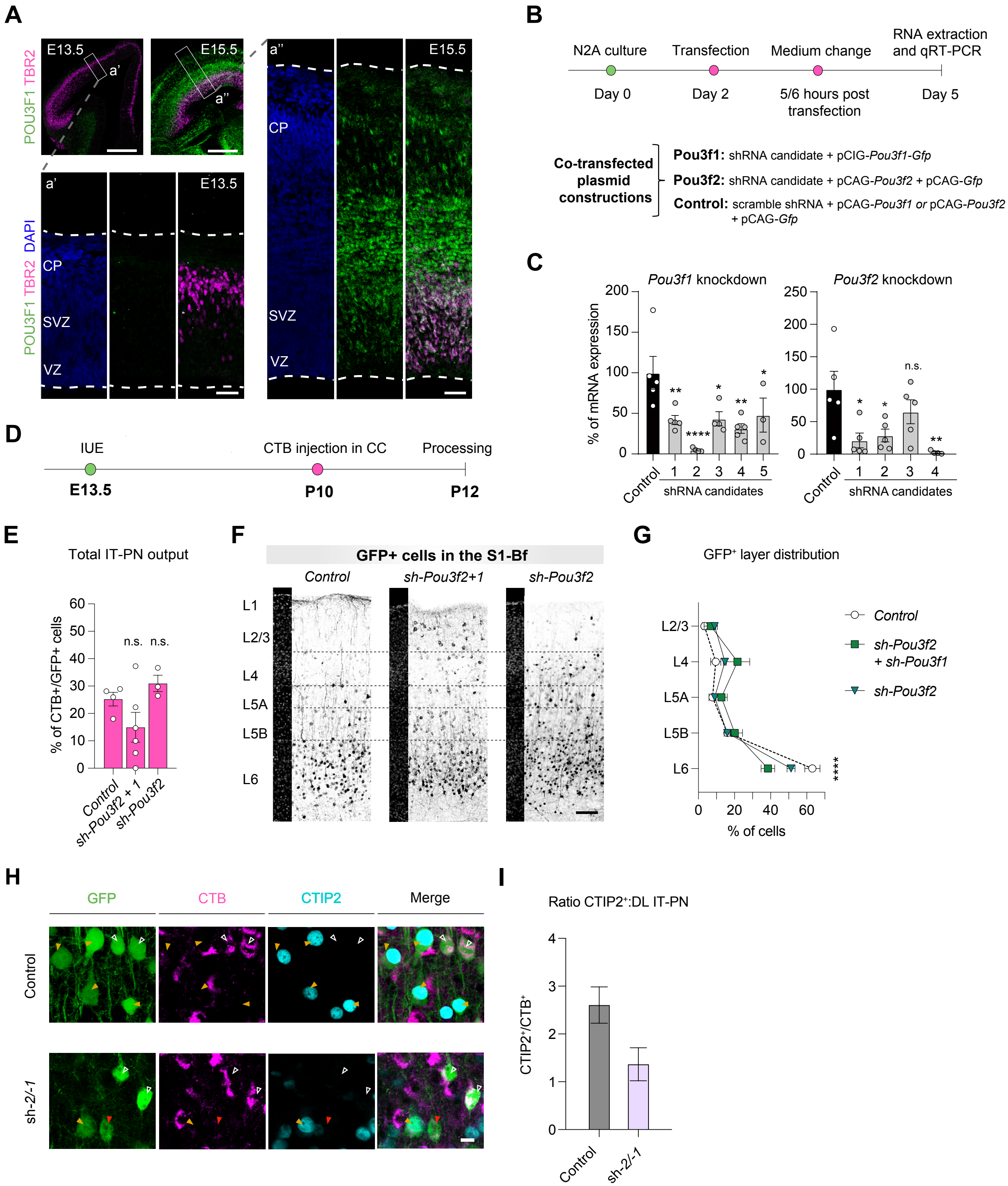
A
E13.5
E15.5
a'
a''
POU3F1 TBR2
POU3F1 TBR2 DAPI
CP
SVZ
VZ
B
N2A culture
Transfection
Medium change
RNA extraction and qRT-PCR
Day 0
Day 2
5/6 hours post transfection
Day 5
Co-transfected plasmid constructions
Pou3f1: shRNA candidate + pCIG-Pou3f1-Gfp
Pou3f2: shRNA candidate + pCAG-Pou3f2 + pCAG-Gfp
Control: scramble shRNA + pCAG-Pou3f1 or pCAG-Pou3f2 + pCAG-Gfp
C
Pou3f1 knockdown
Pou3f2 knockdown
% of mRNA expression
Control
shRNA candidates
n.s.
D
IUE
CTB injection in CC
Processing
E13.5
P10
P12
E
Total IT-PN output
% of CTB+/GFP+ cells
n.s.
Control
sh-Pou3f2 + 1
sh-Pou3f2
F
GFP+ cells in the S1-Bf
Control
sh-Pou3f2+1
sh-Pou3f2
L1
L2/3
L4
L5A
L5B
L6
G
GFP+ layer distribution
Control
sh-Pou3f2 + sh-Pou3f1
sh-Pou3f2
% of cells
H
GFP
CTB
CTIP2
Merge
Control
sh-2/-1
I
Ratio CTIP2+:DL IT-PN
CTIP2+/CTB+
Control
sh-2/-1

Fig. S9.

POU3F1 protein expression and knock-down effects of POU factors in IT-PN production, related to Fig. 6

(**A**) Immunostaining of E13.5 and E15.5 coronal brain slices for POU3F1 (green) and EOMES (magenta). High-magnification areas indicated with white boxes. (**B**) Experimental design for shRNA validation in N2A cells: indicated plasmids were transfected and RNA levels quantified via qRT-PCR. A Scramble shRNA was used as control condition. Five and four candidates were tested against Pou3f1 and Pou3f2, respectively. (**C**) Quantification of mRNA expression in the indicated conditions. Mean ± SEM (n = 5 independent cultures). One-way ANOVA with Dunnnett's post-hoc test: (Pou3f1) ** p-value $_{\text{Scramble vs Sh1}}$ =0.0078, **** p-value $_{\text{Scramble vs Sh2}}$ < 0.0001, * p-value $_{\text{Scramble vs Sh3}}$ =0.0154, ** p-value $_{\text{Scramble vs Sh4}}$ =0.0016, * p-value $_{\text{Scramble vs Sh5}}$ =0.0488, (Pou3f2) * pvalue $_{\text{Scramble vs Sh1}}$ =0.0121, * p-value $_{\text{Scramble vs Sh2}}$ =0.0254, ** p-value $_{\text{Scramble vs Sh4}}$ =0.0033. The most efficient silencers (Sh-2 against *Pou3f1*, Sh-4 against *Pou3f2*) were selected for in vivo experiments based on the degree of mRNA reduction. (**D**) Experimental design. (**E**) Percentage of IT-PNs (CTB^+) over the electroporated population (GFP^+). Percentages were calculated from the total number of CTB^+GFP^+ cells in S1-Bf. Mean ± SEM (n = 2 sections/animal; n ≥ 3 animals/condition). One-way ANOVA with Dunnett's post-hoc test showed non-significant differences. (**F**) GFP signal (black) in the S1-Bf following IUE at E13.5 with the corresponding plasmids. (**G**) Layer distribution of electroporated PNs. Percentages were calculated from the total number of GFP^+ cells in S1-Bf. Mean ± SEM (n = 2 sections/animal; n ≥ 3 animals/condition). Two-way ANOVA with Dunnet's post-hoc test: **** p-value $_{\text{L6 Control vs. L6 sh2/-1}}$ <0.0001. (**H**) Representative images of L5 PNs after E13.5 IUE with CAG-GFP (control) or sh-*Pou3f1* and sh-*Pou3f2* (knockdown) plasmids. GFP is shown in green, CTB in magenta and immunolabeling against CTIP2 in cyan. Yellow arrowheads indicate $CTIP2^+$ electroporated neurons, empty arrowheads show CTIP2-CTB+ electroporated neurons. (**I**) Ratio of $CTIP2^+ET^-$PNs to DL-IT-PNs in control and knockdown electroporated cells. Mean ± SEM (n = 2 sections/animal, n = 3 animals). **Scale bars:** 300 µm (A, low magnification), 30 µm (A, high magnification), 100 µm (F), 10 µm (H).

Data S1. (separate excel file)

Data source and statistics.
Excel files with data and statistics for the main and supplementary figures of this study.

Annex S1.

Atlas of MADM clones traced in this study, related to Figs. 4 and 5

Reconstruction of all MADM clones and subclones in *MADM-11*$^{GT/TG}$; *Emx1-CreERT2* mice with TM-mediated clone induction at E12.5 or E13.5, CTB injection in the CC at P5, and analysis at P10. For each clone, the inferred division pattern, cortical layer distribution, PN-subtype composition, and corresponding clone/subclone classification are shown.

Atlas of MADM clones and subclones

INDEX

1. Proliferative (P) E12.5

2. Proliferative (P) E13.5

3. Asymmetric (A) E12.5

4. Asymmetric (A) E13.5

5. Terminal (T) E12.5

6. Terminal (T) E13.5

7. Orphan (O) E12.3 and E13.5

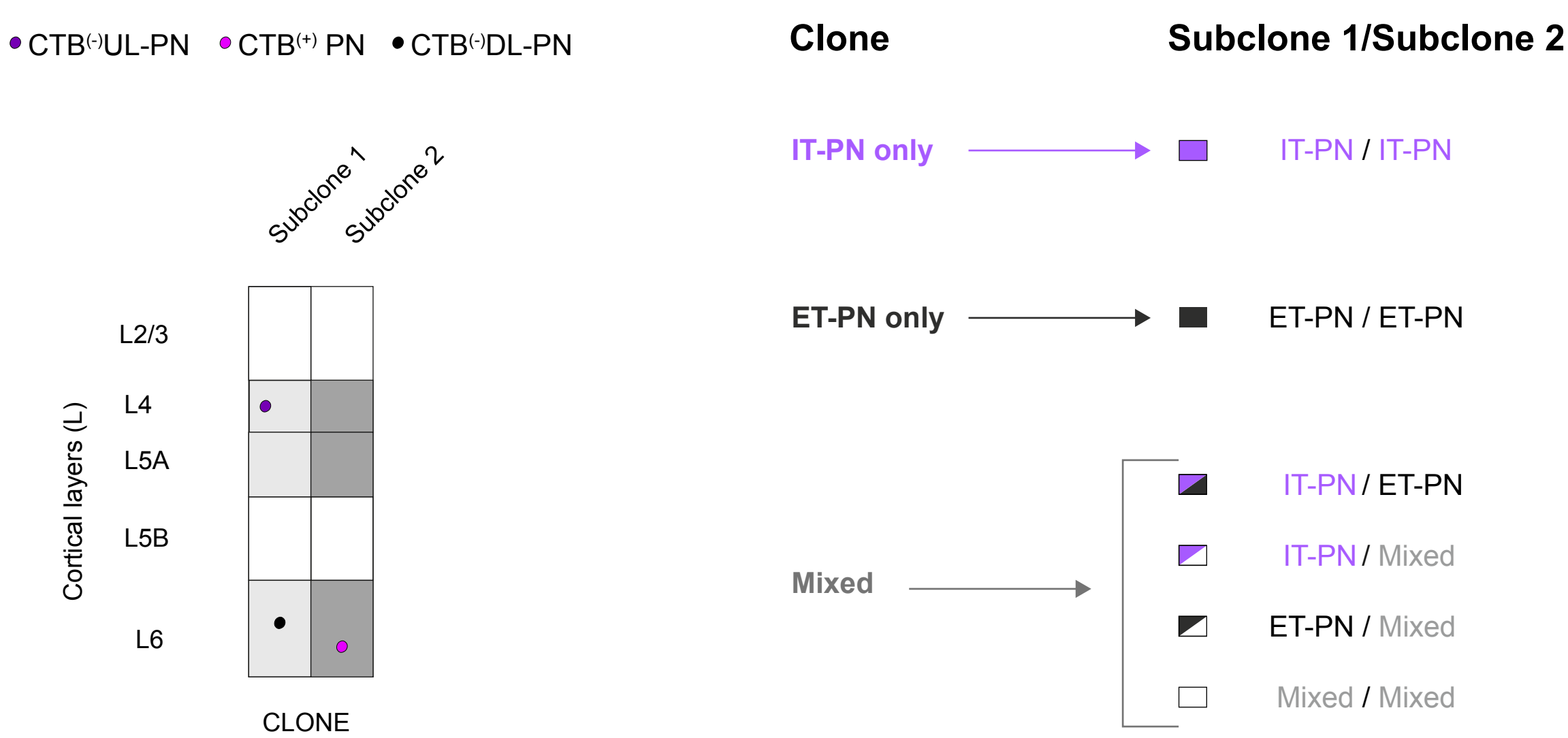

Proliferative clones E12.5: • CTB(-)UL-PN • CTB(+) PN • CTB(-)DL-PN

A

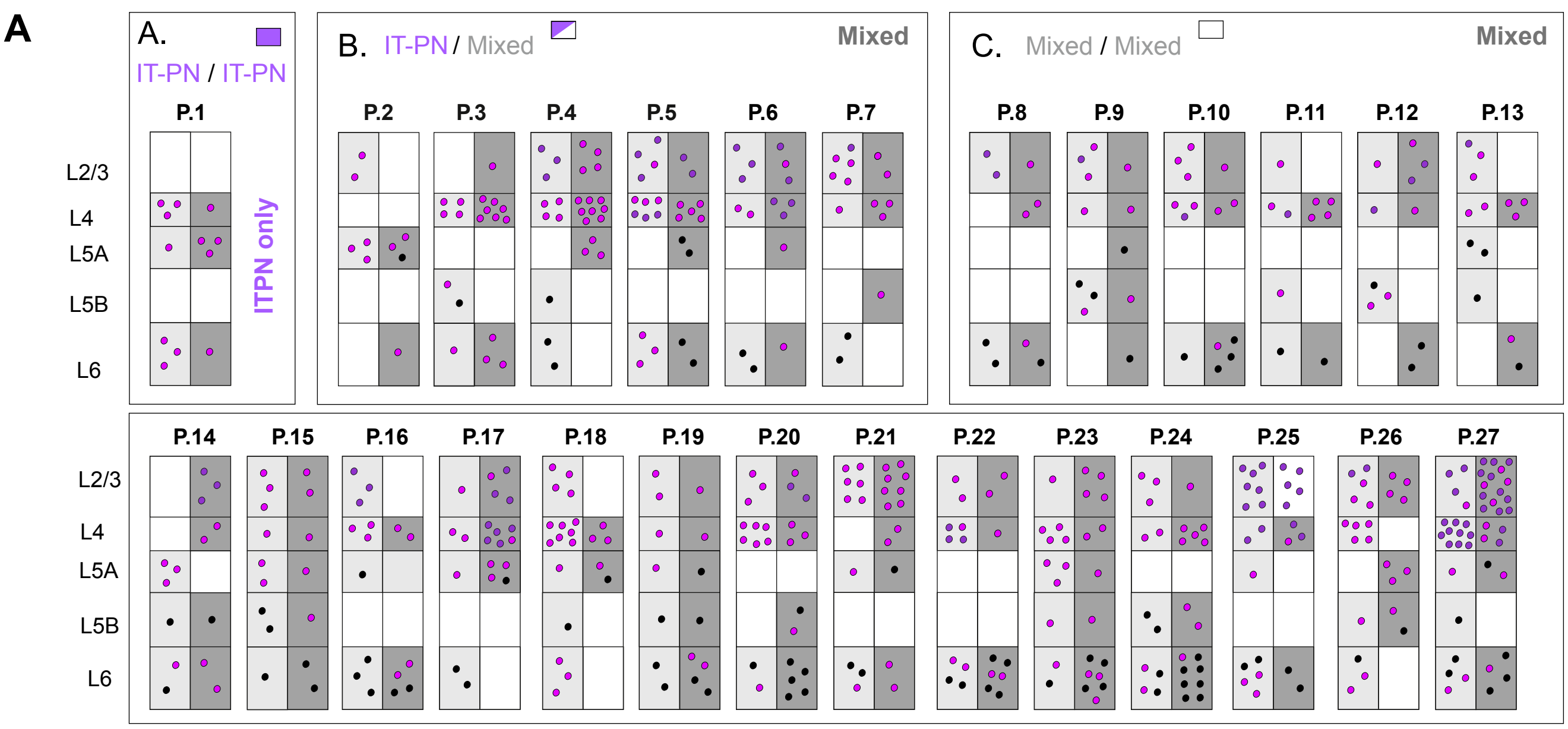

B

E12.5	Subclone.1	Subclone.2 (Proliferative clones)	Size .1	Size .2	Total Size
**A.** (IT-PN/IT-PN)					
P.1	L6 L5A L5A L5A L4	L6 L6 L6 L5A L4 L4 L4	5	7	12
**B.** (IT-PN/Mixed)					
P.2	L6 L5A L5A L5A	L5A L5A L5A L2/3 L2/3	4	5	9
P.3	L5B L6 L5B L4 L4 L4 L4	L6 L6 L6 L4 L4 L4 L4 L4 L4 L4 L2/3	7	11	18
P.4	L6 L6 L5B L4 L4 L4 L4 L2/3 L2/3 L2/3	L5A L5A L5A L4 L4 L4 L4 L4 L4 L4 L4 L2/3 L2/3 L2/3 L2/3	10	15	25
P.5	L6 L6 L5A L5A L4 L4 L4 L4 L4 L2/3 L2/3	L6 L6 L6 L4 L4 L4 L4 L4 L4 L2/3 L2/3 L2/3 L2/3	11	13	24
P.6	L6 L6 L4 L4 L2/3 L2/3 L2/3	L6 L5A L4 L4 L4 L2/3 L2/3 L2/3	7	8	15
P.7	L6 L6 L4 L2/3 L2/3 L2/3 L2/3 L2/3	L5B L4 L4 L4 L2/3 L2/3	8	6	14
**C.** (Mixed/Mixed)					
P.8	L6 L6 L2/3 L2/3	L6 L6 L4 L4 L2/3	4	5	9
P.9	L6 L5A L5B L4 L2/3	L5B L5B L5B L4 L2/3 L2/3 L2/3	5	7	12
P.10	L6 L4 L4 L4 L2/3 L2/3 L2/3	L6 L6 L6 L6 L4 L4 L2/3	7	7	14
P.11	L6 L5B L4 L4 L2/3	L6 L4 L4 L4 L4	5	5	10
P.12	L5B L5B L5B L4 L2/3	L6 L6 L4 L2/3 L2/3 L2/3	5	6	11
P.13	L6 L6 L4 L4 L4	L5B L5A L5A L4 L4 L2/3 L2/3 L2/3	5	8	13
P.14	L6 L5B L6 L5A L5A L5A	L5B L6 L6 L4 L4 L2/3 L2/3 L2/3	6	8	14
P.15	L6 L6 L5B L5A L4 L2/3 L2/3	L6 L5B L5B L5A L5A L4 L2/3 L2/3 L2/3	7	9	16
P.16	L6 L6 L6 L6 L4 L4	L6 L6 L6 L5A L4 L4 L4 L2/3 L2/3 L2/3	6	10	16
P.17	L6 L6 L5A L4 L4 L2/3	L5A L5A L5A L5A L4 L4 L4 L4 L4 L4 L2/3 L2/3 L2/3 L2/3	6	14	20
P.18	L5A L5A L4 L4 L4	L5B L6 L6 L6 L5A L4 L4 L4 L4 L4 L4 L2/3 L2/3 L2/3 L2/3	5	16	21
P.19	L6 L6 L5B L5A L4 L2/3 L2/3	L6 L6 L5B L6 L6 L5A L4 L2/3	7	8	21
P.20	L6 L6 L4 L4 L4 L4 L4 L4 L2/2 L2/2 L2/3	L6 L6 L6 L6 L6 L5B L5B L4 L4 L4 L2/3 L2/3 L2/3	11	13	24
P.21	L6 L6 L6 L5A L2/3 L2/3 L2/3 L2/3 L2/3 L2/3	L6 L6 L5A L4 L4 L2/3 L2/3 L2/3 L2/3 L2/3 L2/3 L2/3 L2/3	10	13	23
P.22	L6 L6 L6 L6 L6 L6 L4 L2/3 L2/3	L6 L6 L6 L6 L4 L4 L4 L4 L2/3 L2/3	9	10	19
P.23	L6 L6 L5B L5A L5A L5A L4 L4 L4 L4 L2/3	L6 L6 L6 L6 L6 L6 L6 L5B L5A L4 L4 L2/3 L2/3 L2/3 L2/3	11	15	26
P.24	L6 L6 L5B L5B L6 L6 L4 L4 L2/3 L2/3 L2/3	L6 L6 L6 L6 L6 L6 L6 L6 L5B L5B L4 L4 L4 L4 L4 L2/3	11	16	27
P.25	L6 L6 L4 L4 L4 L2/3 L2/3 L2/3 L2/3 L2/3	L6 L6 L6 L6 L6 L5A L4 L4 L2/3 L2/3 L2/3 L2/3 L2/3 L2/3 L2/3	10	15	25
P.26	L5B L5B L5A L5A L5A L2/3 L2/3 L2/3 L2/3	L6 L6 L6 L5B L4 L4 L4 L4 L4 L2/3 L2/3 L2/3 L2/3 L2/3	9	14	23
P.27	L6 L6 L5B L6 L6 L5A L4 L4 L4 L4 L4 L4 L4 L4 L4 L4 L2/3 L2/3 L2/3 L2/3	L6 L6 L6 L6 L5A L5A L4 L4 L4 L4 L2/3 L2/3 L2/3 L2/3 L2/3 L2/3 L2/3 L2/3 L2/3 L2/3 L2/3 L2/3 L2/3 L2/3 L2/3	20	25	45

Proliferative clones E13.5: • CTB$^{(-)}$UL-PN • CTB$^{(+)}$ PN • CTB$^{(-)}$DL-PN

A

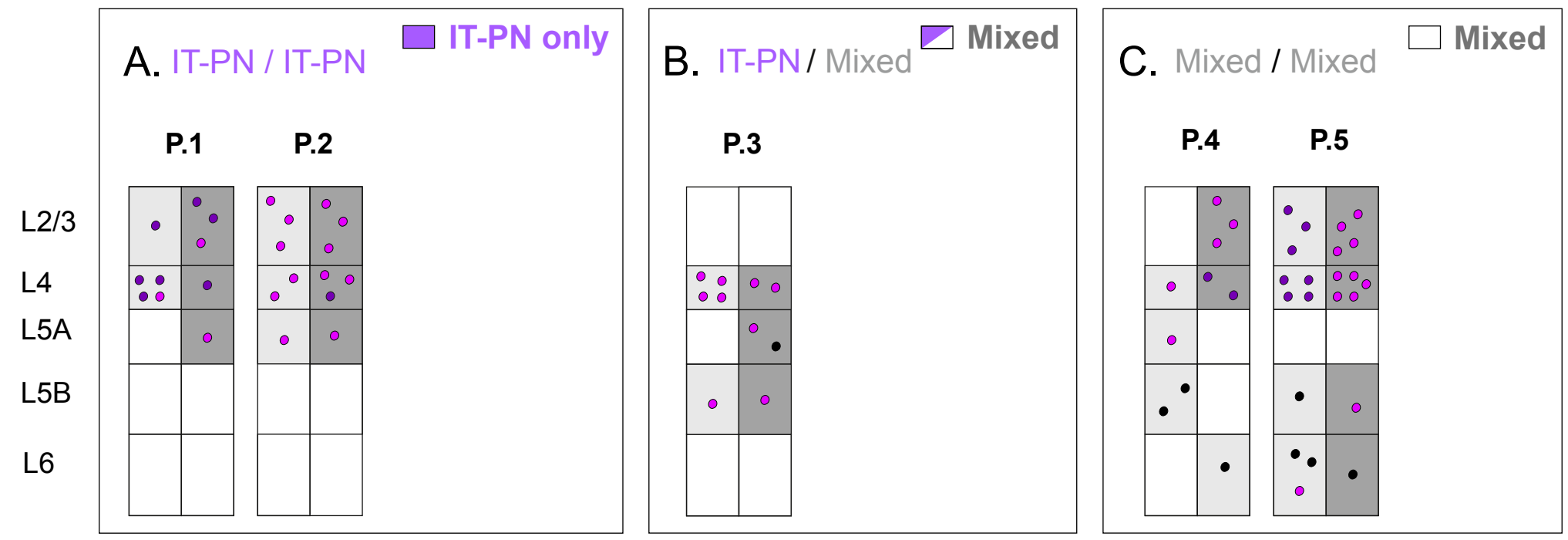

B

E13.5		Proliferative clones: Subclone 1	Subclone 2	Size .1	Size .2	Total Size
A. IT-PN/IT-PN	P.1	L4 L4 L4 L4 L2/3	L5A L4 L2/3 L2/3 L2/3	5	5	10
	P.2	L5A L4 L4 L2/3 L2/3 L2/3	L5A L4 L4 L4 L2/3 L2/3 L2/3	6	7	13
B. IT-PN/Mixed	P.4	L5B L4 L4 L4 L4	L5B L5A L5A L4 L4	4	6	10
C. Mixed/Mixed	P.4	L5B L5B L5A L4	L6 L4 L4 L2/3 L2/3 L2/3	4	6	10
	P.5	L6 L6 L6 L5B L4 L4 L4 L4 L2/3 L2/3 L2/3	L6 L5B L4 L4 L4 L4 L4 L2/3 L2/3 L2/3 L2/3	6	7	13

Asymmetric clones E12.5: • CTB(-)UL-PN • CTB(+) PN • CTB(-)DL-PN

A

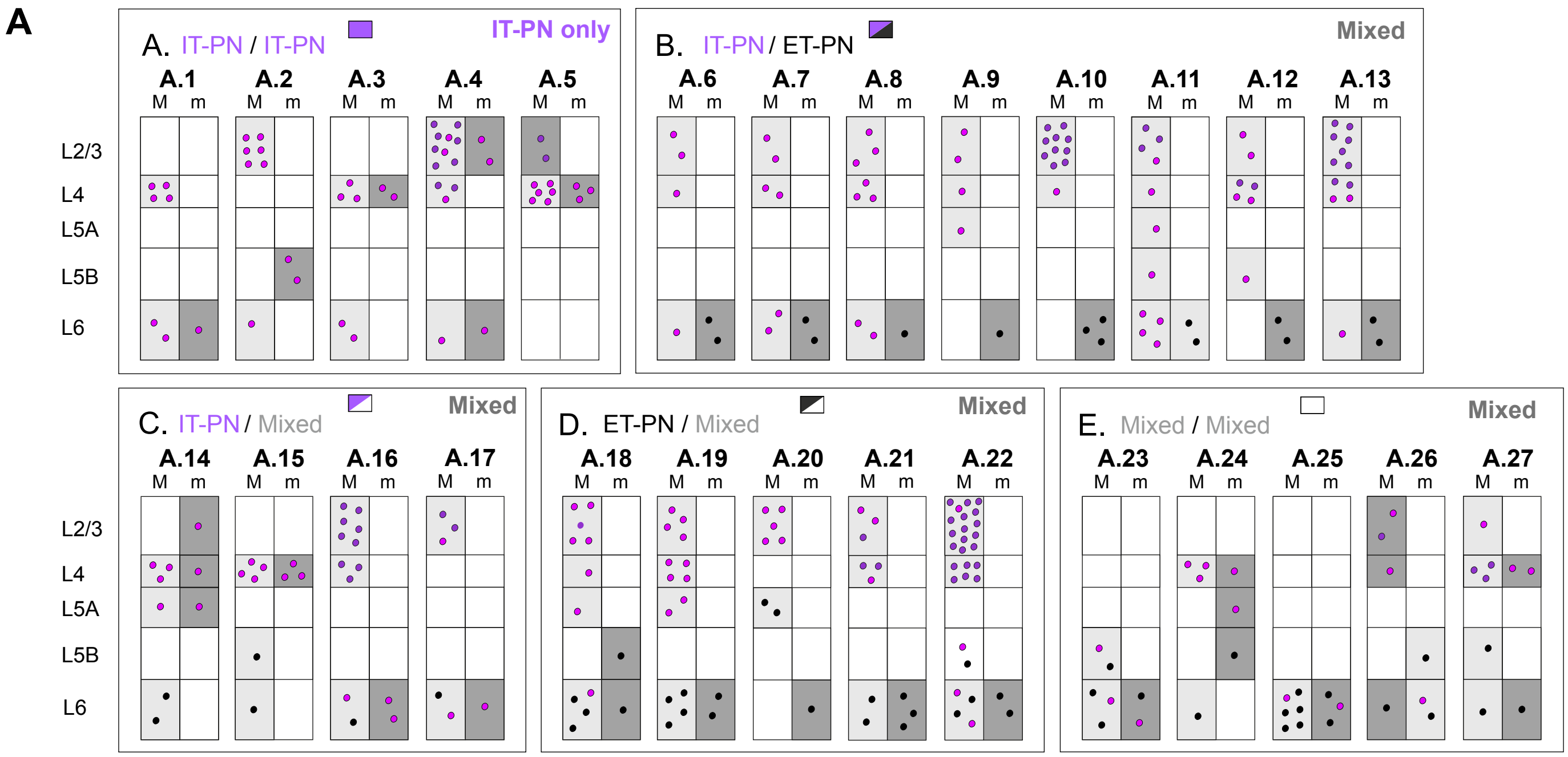

B

E12.5		minority (m)	MAJORITY (M) Asymmetric clones	Size m	Size M	Size
A. IT-PN/IT-PN	A.1	L6	L6 L6 L4 L4 L4 L4	1	6	7
	A.2	L5B L5B	L6 L2/3 L2/3 L2/3 L2/3 L2/3 L2/3	2	7	9
	A.3	L4 L4	L6 L6 L4 L4 L4	2	5	7
	A.4	L6 L2/3 L2/3	L6 L4 L4 L4 L2/3 L2/3 L2/3 L2/3 L2/3 L2/3 L2/3 L2/3 L2/3	3	13	16
	A.5	L4 L4 L4	L4 L4 L4 L4 L4 L4 L2/3 L2/3	3	8	11
B. ITP-N/ET-PN	A.6	L6 L6	L6 L4 L2/3 L2/3	2	4	6
	A.7	L6 L6	L6 L6 L4 L4 L2/3 L2/3	2	6	8
	A.8	L6	L6 L6 L4 L4 L4 L2/3 L2/3 L2/3	1	8	9
	A.9	L6	L5A L4 L2/3 L2/3	1	4	5
	A.10	L6 L6 L6	L4 L2/3 L2/3 L2/3 L2/3 L2/3 L2/3 L2/3 L2/3 L2/3 L2/3	3	11	14
	A.11	L6 L6	L6 L6 L6 L6 L5B L5A L4 L2/3 L2/3 L2/3 L2/3	2	11	13
	A.12	L6 L6	L5B L4 L4 L4 L4 L2/3 L2/3	2	7	9
	A.13	L6 L6	L6 L4 L4 L4 L4 L2/3 L2/3 L2/3 L2/3 L2/3 L2/3 L2/3	2	12	14
C. IT-PN/Mixed	A.14	L5A L4 L2/3	L6 L6 L5A L4 L4 L4	3	6	9
	A.15	L4 L4 L4	L6 L5B L4 L4 L4 L4	3	6	9
	A.16	L6 L6	L6 L6 L4 L4 L4 L2/3 L2/3 L2/3 L2/3 L2/3 L2/3	2	11	13
	A.17	L6	L6 L6 L2/3 L2/3 L2/3	1	5	6
D. ET-PN/Mixed	A.18	L6 L5B	L6 L6 L6 L6 L5A L4 L2/3 L2/3 L2/3 L2/3 L2/3 L2/3	2	11	13
	A.19	L6 L6	L6 L6 L6 L6 L5A L5A L4 L4 L4 L4 L2/3 L2/3 L2/3 L2/3	2	14	16
	A.20	L6	L5A L5A L2/3 L2/3 L2/3 L2/3 L2/3	1	7	8
	A.21	L6 L6 L6	L6 L6 L4 L4 L4 L2/3 L2/3 L2/3	3	8	11
	A.22	L6 L6	L6 L6 L5B L6 L6 L5B L4 L4 L4 L4 L4 L4 L2/3 L2/3 L2/3 L2/3 L2/3 L2/3 L2/3 L2/3 L2/3 L2/3 L2/3 L2/3 L2/3 L2/3 L2/3	2	27	29
E. Mixed/Mixed	A.23	L6 L6	L6 L6 L5B L6 L5B	2	5	7
	A.24	L5B L5A L4	L6 L4 L4 L4	3	4	7
	A.25	L6 L6 L6	L6 L6 L6 L6 L6 L6	3	6	9
	A.26	L6 L5B L6	L6 L4 L2/3 L2/3	3	4	7
	A.27	L6 L4 L4	L6 L5B L4 L4 L4 L2/3	3	6	9

Asymmetric clones E13.5: • CTB(-)UL-PN • CTB(+) PN • CTB(-)DL-PN

A

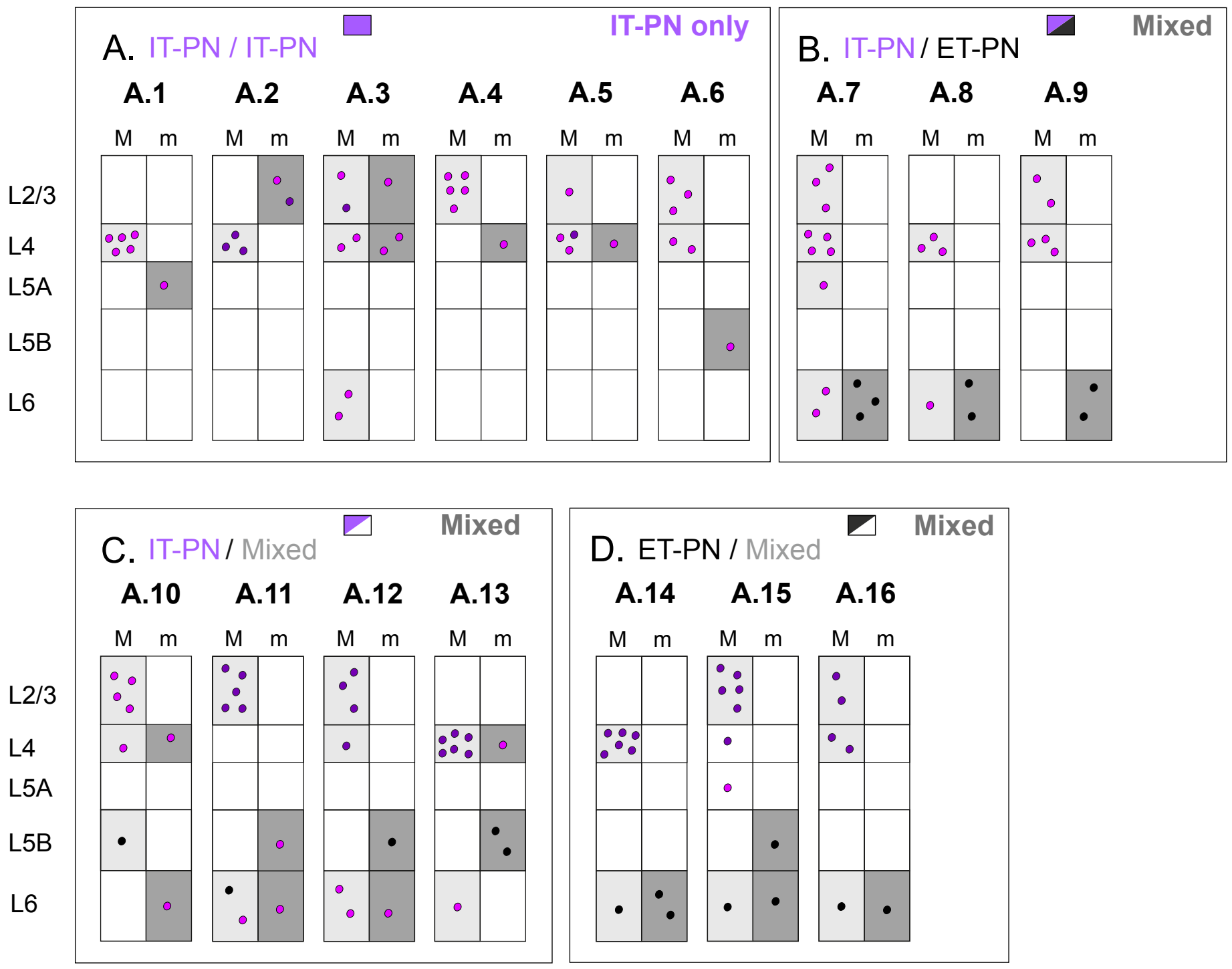

B

E13.5		minority (m)	MAJORITY (M) — Asymmetric clones	Size m	Size M	Size
A. IT-PN/IT-PN	A.1	L5A	L4 L4 L4 L4 L4	1	5	6
	A.2	L2/3 L2/3	L4 L4 L4 L2/3 L2/3	2	5	7
	A.3	L4 L4 L2/3	L6 L6 L4 L4 L2/3 L2/3	3	6	9
	A.4	L4	L2/3 L2/3 L2/3 L2/3 L2/3	1	5	6
	A.5	L4	L4 L4 L4 L2/3	1	4	5
	A.6	L5B	L4 L4 L2/3 L2/3 L2/3	1	5	6
B. IT-PN/ET-PN	A.7	L6 L6 L6	L6 L6 L5A L4 L4 L4 L4 L2/3 L2/3 L2/3	3	10	13
	A.8	L6 L6	L6 L4 L4 L4	2	4	6
	A.9	L6	L4 L4 L4 L2/3 L2/3	1	5	6
C. IT-PN/Mixed	A.10	L4 L6	L5B L4 L2/3 L2/3 L2/3 L2/3	2	6	8
	A.11	L6 L5B	L6 L6 L2/3 L2/3 L2/3 L2/3 L2/3 L2/3	2	8	10
	A.12	L5B L6	L6 L6 L4 L2/3 L2/3 L2/3	2	6	8
	A.13	L5B L5B L4	L6 L4 L4 L4 L4 L4 L4	3	7	10
D. ET-PN/Mixed	A.14	L6 L6	L6 L4 L4 L4 L4 L4 L4	2	7	9
	A.15	L6 L5B	L6 L5A L4 L2/3 L2/3 L2/3 L2/3 L2/3	2	8	10
	A.16	L6	L6 L4 L4 L2/3 L2/3	1	5	6

Terminal clones E12.5: • CTB(-) UL-PN • CTB(+) PN • CTB(-) DL-PN

A

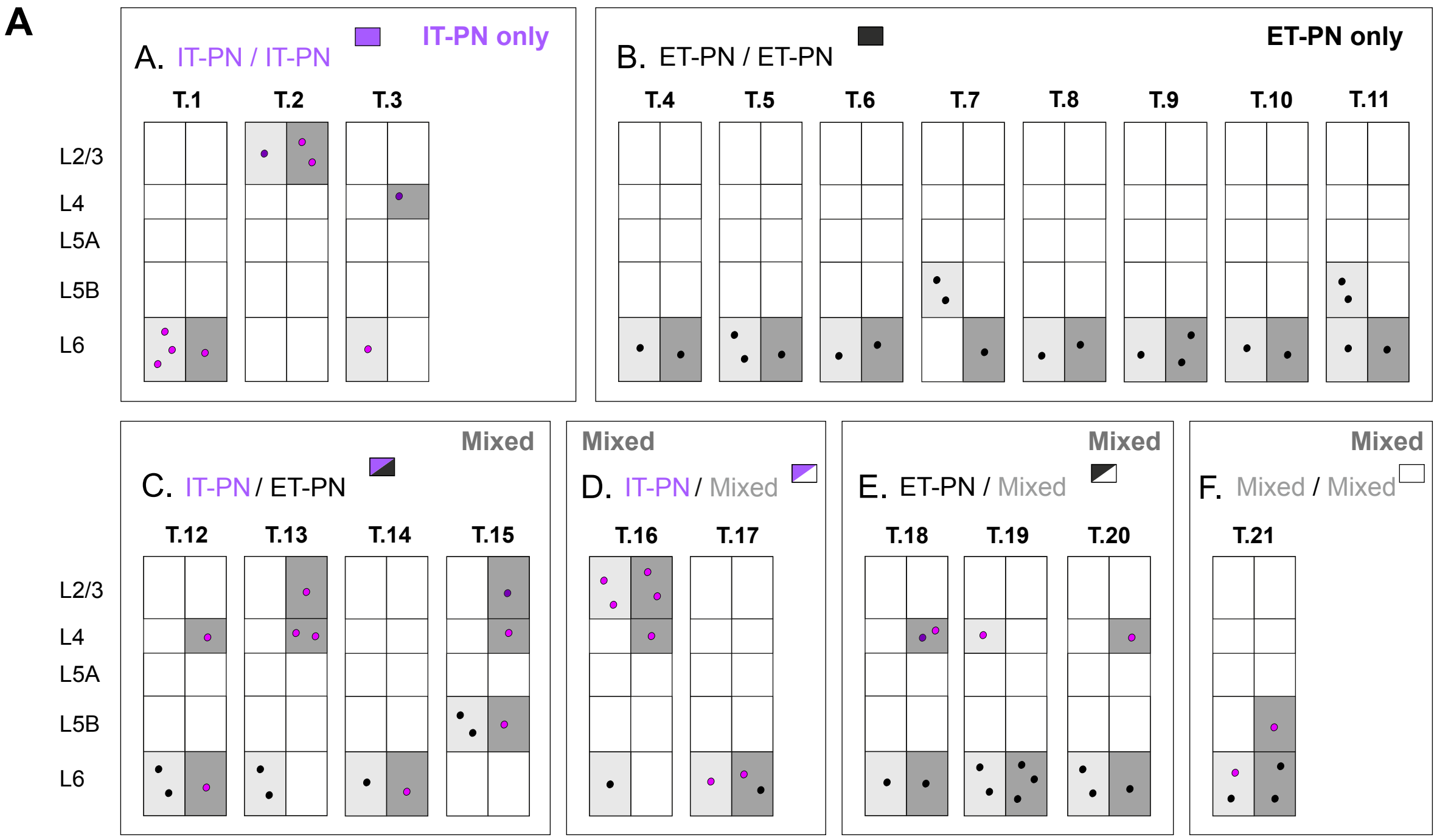

B

E12.5		Subclone 1	Subclone 2	Terminal clones	Size .1	Size .2	Size
A. IT-PN/IT-PN	T.1	L6 L6 L6	L6		3	1	4
	T.2	L2/3	L2/3 L2/3		1	2	3
	T.3	L6	L4 L4		1	2	3
B. ET-PN/ET-PN	T.4	L6	L6		1	1	2
	T.5	L6 L6	L6		2	1	3
	T.6	L5B L5B	L6		2	1	3
	T.7	L6	L6		1	1	2
	T.8	L6	L6 L6		1	2	3
	T.9	L6	L6		1	1	2
	T.10	L6	L6		1	1	2
	T.11	L6 L5B L5B	L6		3	1	4
C. IT-PN/ET-PN	T.12	L6 L6	L6 L4		2	2	4
	T.13	L6 L6	L4 L4 L2/3		2	3	5
	T.14	L6	L6		1	1	2
	T.15	L5B L5B	L5B L4 L2/3		2	3	5
D. IT-PN/Mixed	T.16	L4 L2/3 L2/3	L6 L2/3 L2/3		3	3	6
	T.17	L6	L6 L6		1	2	3
E. ET-PN/Mixed	T.18	L6	L6 L4 L4		1	3	4
	T.19	L6 L6 L6	L6 L6 L4		3	3	6
	T.20	L6 L6	L6 L4		2	2	4
F. Mixed/Mixed	T.21	L6 L6	L6 L6 L5B		2	3	5

Terminal clones E13.5: • CTB$^{(-)}$UL-PN • CTB$^{(+)}$ PN • CTB$^{(-)}$DL-PN

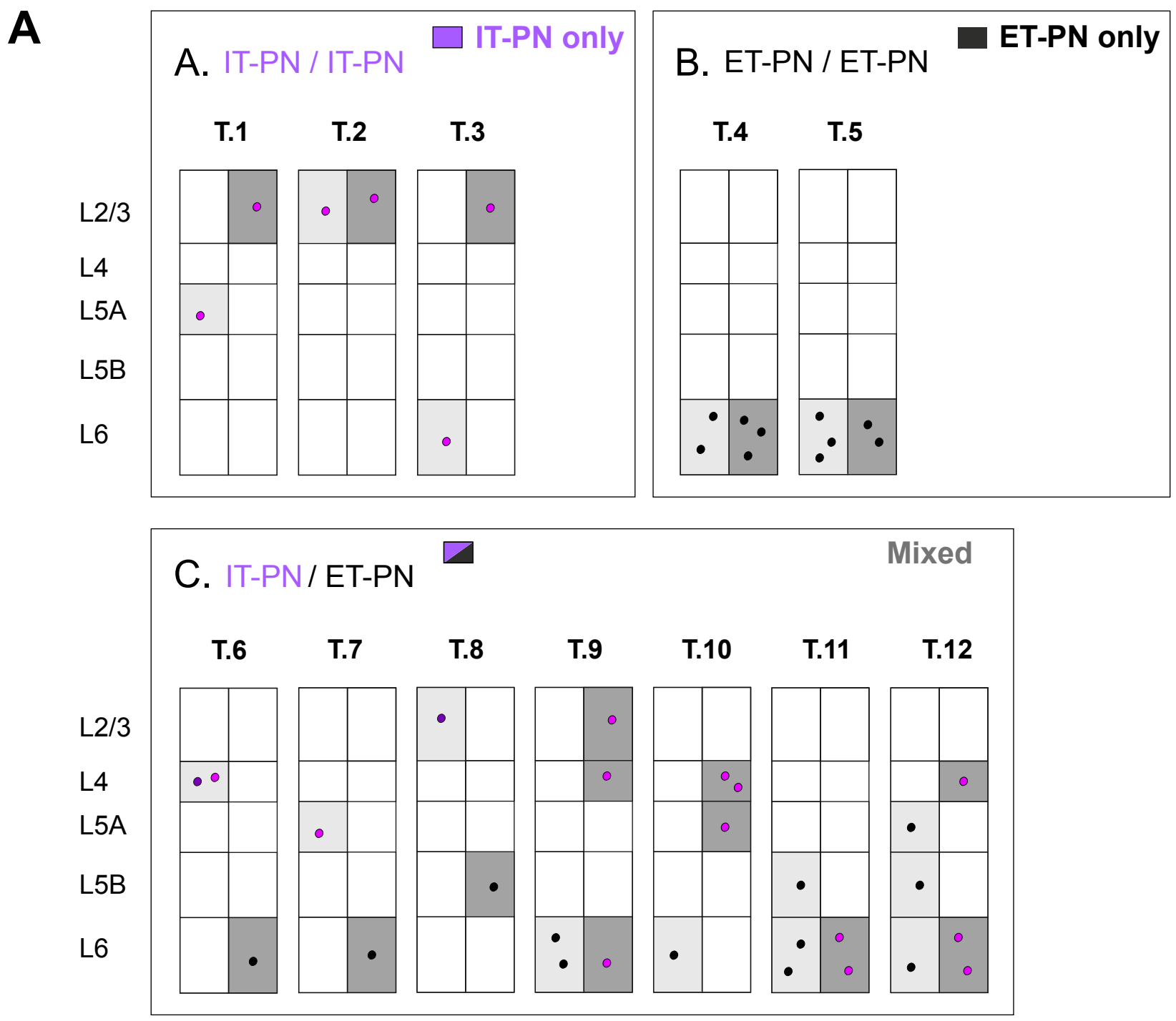

B

	E13.5	Subclone1	Subclone 2	Size .1	Size .2	Size
A. IT-PN/IT-PN	T.1	L5A	L2/3	1	1	2
	T.2	L2/3	L2/3	1	1	2
	T.3	L6	L2/3	1	1	2
B. ET-PN/ET-PN	T.4	L6 L6	L6 L6 L6	2	3	5
	T.5	L6 L6 L6	L6 L6	3	2	5
C. IT-PN/ET-PN	T.6	L4 L4	L6	2	1	3
	T.7	L5A	L6	1	1	2
	T.8	L2/3	L5B	1	1	2
	T.9	L6 L4 L2/3	L6 L6	3	2	5
	T.10	L5A L4 L4	L6	3	1	4
	T.11	L6 L6	L6 L6 L5B	2	3	5
	T.12	L6 L6 L4	L6 L5B L5A	3	3	6

Terminal clones

Orphan clones E12.5: • CTB(-)UL-PN • CTB(+) PN • CTB(-)DL-PN

A

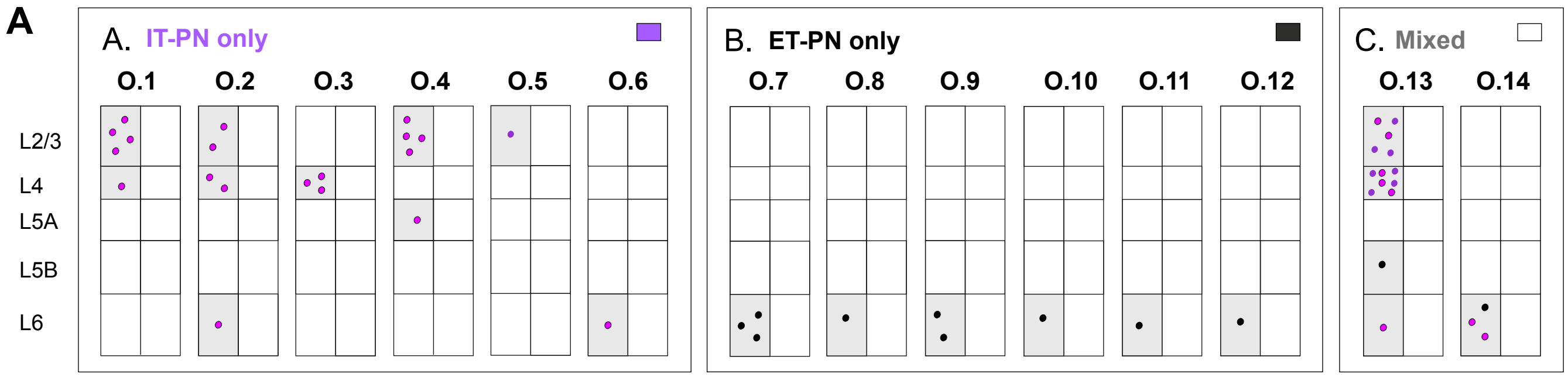

B **E12.5**

		Orphan clones	Size
A. (ITPN)	O.1	L4 L2/3 L2/3 L2/3 L2/3	5
	O.2	L6 L4 L4 L2/3 L2/3	5
	O.3	L4 L4 L4	3
	O.4	L5A L2/3 L2/3 L2/3 L2/3	5
	O.5	L2/3	1
	O.6	L6	1
B. (ETPN)	O.7	L6 L6 L6	3
	O.8	L6	1
	O.9	L6 L6	2
	O.10	L6	1
	O.11	L6	1
	O.12	L6	1
C. (Mixed)	O.13	L6 L5B L4 L4 L4 L4 L4 L4 L4 L2/3 L2/3 L2/3 L2/3 L2/3	14
	O.14	L6 L6 L6	3

C **E13.5**

		Orphan clones	Size
A. (ITPN)	O.1	L4 L2/3 L2/3	3
	O.2	L6	1
	O.3	L4 L2/3	2
	O.4	L4 L2/3 L2/3 L2/3	4
	O.5	L4	1
	O.6	L2/3	1
	O.7	L4 L2/3	2
	O.8	L2/3 L2/3 L2/3 L2/3 L2/3 L2/3 L2/3 L2/3	8
	O.9	L4 L4	2
	O.10	L2/3	1
	O.11	L4 L4 L4 L2/3 L2/3	5
	O.12	L5A L4 L2/3 L2/3 L2/3	5
	O.13	L2/3	1
	O.14	L6 L5A L4	3
B. (ETPN)	O.15	L5A	1
	O.16	L5A	1

Orphan clones E13 .5: • CTB(-)UL-PN • CTB(+) PN • CTB(-)DL-PN

D

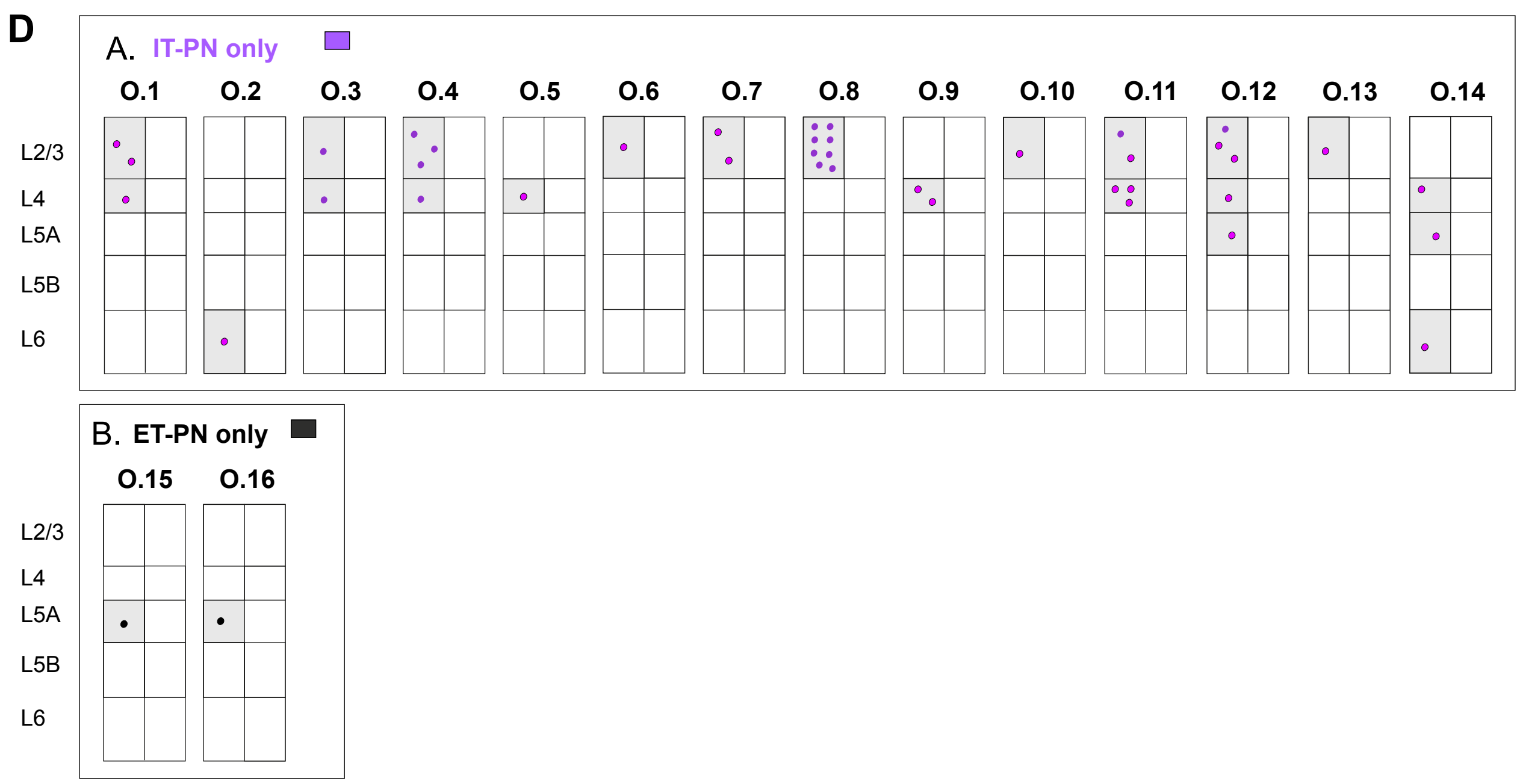

Supplement: Supplementary file 1 [file sciadv.adw5487_sm.pdf]
